# Oxazole-Bridged Combretastatin A-4 Derivatives with Tethered Hydroxamic Acids: Structure–Activity Relations of New Inhibitors of HDAC and/or Tubulin Function

**DOI:** 10.3390/ijms20020383

**Published:** 2019-01-17

**Authors:** Florian Schmitt, Lisa Chiara Gosch, Alexandra Dittmer, Matthias Rothemund, Thomas Mueller, Rainer Schobert, Bernhard Biersack, Andrea Volkamer, Michael Höpfner

**Affiliations:** 1 Department of Chemistry, University of Bayreuth, Universitaetsstrasse 30, 95447 Bayreuth, Germany; florian1.schmitt@uni-bayreuth.de (F.S.); matthias.rothemund@uni-bayreuth.de (M.R.); rainer.schobert@uni-bayreuth.de (R.S.); 2 Institute of Physiology, Charité–Universitätsmedizin Berlin, Charitéplatz 1, 10117 Berlin, Germany; lisa-chiara.gosch@charite.de (L.C.G.); alexandra.dittmer@charite.de (A.D.); 3 In Silico Toxicology Group, Institute of Physiology, Charité–Universitätsmedizin Berlin, Charitéplatz 1, 10117 Berlin, Germany; andrea.volkamer@charite.de; 4 Internal Medicine IV, University Hospital Halle (Saale), Ernst-Grube-Str. 40, 06120 Halle, Germany; thomas.mueller@medizin.uni-halle.de

**Keywords:** combretastatin A-4, oxazole, histone deacetylase, tubulin, anticancer agents

## Abstract

New inhibitors of tubulin polymerization and/or histone deacetylase (HDAC) activity were synthesized by attaching alkyl tethered hydroxamic acid appendages of varying length to oxazole-bridged combretastatin A-4 analogous caps. While their antiproliferative and microtubule disrupting effect was most pronounced for derivatives with short spacers, HDAC inhibition was strongest for those with longer spacers. These findings were further supported by computational methods such as structure-based docking experiments exploring the target interactions of the derivatives with varying linkers. For instance, compounds featuring short four-atom spacers between cap and hydroxamic acid inhibited the growth of various cancer cell lines and human endothelial hybrid cells with IC_50_ values in the low nanomolar range. In line with their ability to inhibit the microtubule assembly, four- and five-atom spacered hydroxamic acids caused an accumulation of 518A2 melanoma cells in G2/M phase, whereas a compound featuring a six-atom spacer and performing best in HDAC inhibition, induced a G1 arrest in these cells. All these beneficial anticancer activities together with their selectivity for cancer cells over non-malignant cells, point out the great potential of these novel pleiotropic HDAC and tubulin inhibitors as drug candidates for cancer therapy.

## 1. Introduction

**Figure 1:**
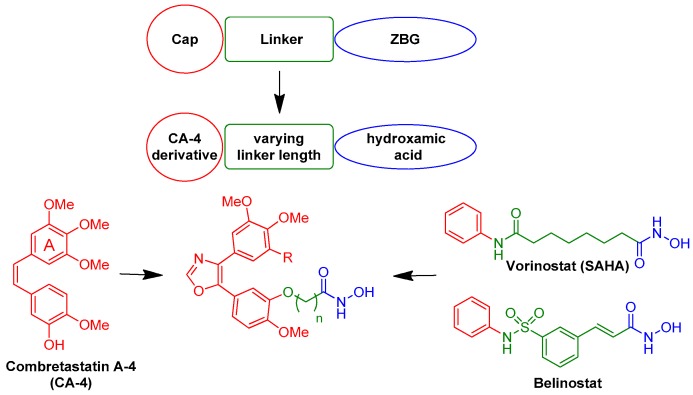
General pharmacophore model of HDACi, structures of the vascular-disrupting agent (VDA) combretastatin A-4 (CA-4) and of the HDACi vorinostat (SAHA) and belinostat as well as the combination of CA-4 derivatives and HDACi in our hybrid compounds.

Histone deacetylases (HDAC) catalyze the deacetylation of ε-*N*-acetyl lysine residues of histones thus regulating the expression of genes which are important for crucial cellular processes such as chromatin condensation and decondensation (DNA replication, transcription, and repair). Certain HDAC enzymes also modify non-histone proteins such as signal transduction mediators, transcription factors and regulators, as well as structural proteins resulting in modulation of cell growth, differentiation, migration, and angiogenesis [1]. HDACs are overexpressed in various solid tumors, e.g., in gastric cancer, prostate cancer, breast cancer, and colorectal cancer [2,3,4,5,6]. HDAC of class I (HDAC1, 2, 3, and 8), class IIa (HDAC4, 5, 7, and 9), class IIb (HDAC6, and 10) and class IV (HDAC11) share a zinc(II) cation in the center of their catalytic cavity which is the target of several approved HDAC inhibitors (HDACi) [1]. By the development of several HDACi over the last two decades, a robust pharmacophore model for zinc-dependent HDACi was established typically consisting of a zinc binding group (ZBG), a linker, and a capping group (Figure 1). These HDACi mimic the natural substrate acetyl-lysine and exert their effect by coordination of the zinc(II) center with ligands such as benzamides, carboxylates, or hydroxamic acids [7]. The ZBGs should be connected to the cap by a hydrophobic linker, which is slim enough to fit in the tunnel between the active site and the capping groups. The latter are used for surface recognition and can induce sub-class selectivity [8,9]. Several HDACi such as vorinostat (SAHA, Figure 1), belinostat (PXD101, Figure 1) and panobinostat (LBH589) which were modelled on this pharmacophore template are already approved for the therapy of lymphoma and myeloma [10,11,12,13]. Several other HDACi are recently under clinical investigation, since HDACi of the first generation have shown certain shortcomings in solid tumors such as induction of epithelial-to-mesenchymal transition (EMT) in prostate cancer cells [14,15,16]. In order to overcome such drawbacks, HDACi with dual or multimodal activities including kinase inhibition or DNA alkylation/metalation were introduced [17].

Microtubules are vital components of the cytoskeleton and thus an important target in cancer chemotherapy [18,19]. Interestingly, HDACi have shown synergetic effects when combined with tubulin-binding anticancer drugs [20,21,22]. Thus, HDACi harboring tubulin-targeting structural motifs appear to be promising anticancer drug candidates [23,24,25]. While colchicine- and quinazoline-based dual inhibitors were already published, no dual inhibitors based on the potent microtubule disrupting agent (MDA) combretastatin A-4 (CA-4, Figure 1) are known, so far. Herein, we present a new series of tubulin-targeting oxazole-bridged CA-4 derivatives with hydroxamate appendages. We chose the oxazole-bridged CA-4 scaffold because of its improved stability when compared with the *cis*-stilbene CA-4 parent compound [26,27]. We investigated how the linker length of the new hybrid compounds affects the compounds’ potency to inhibit HDAC1 and HDAC6, as well as the microtubule assembly. Moreover, the anti-proliferative, anti-migratory, and further anticancer activities of the new hybrid compounds were evaluated, and computational methods were used to predict and to explain binding modes and affinities of the studied compounds. 

## 2. Results

### 2.1. Chemistry

**Scheme 1:**
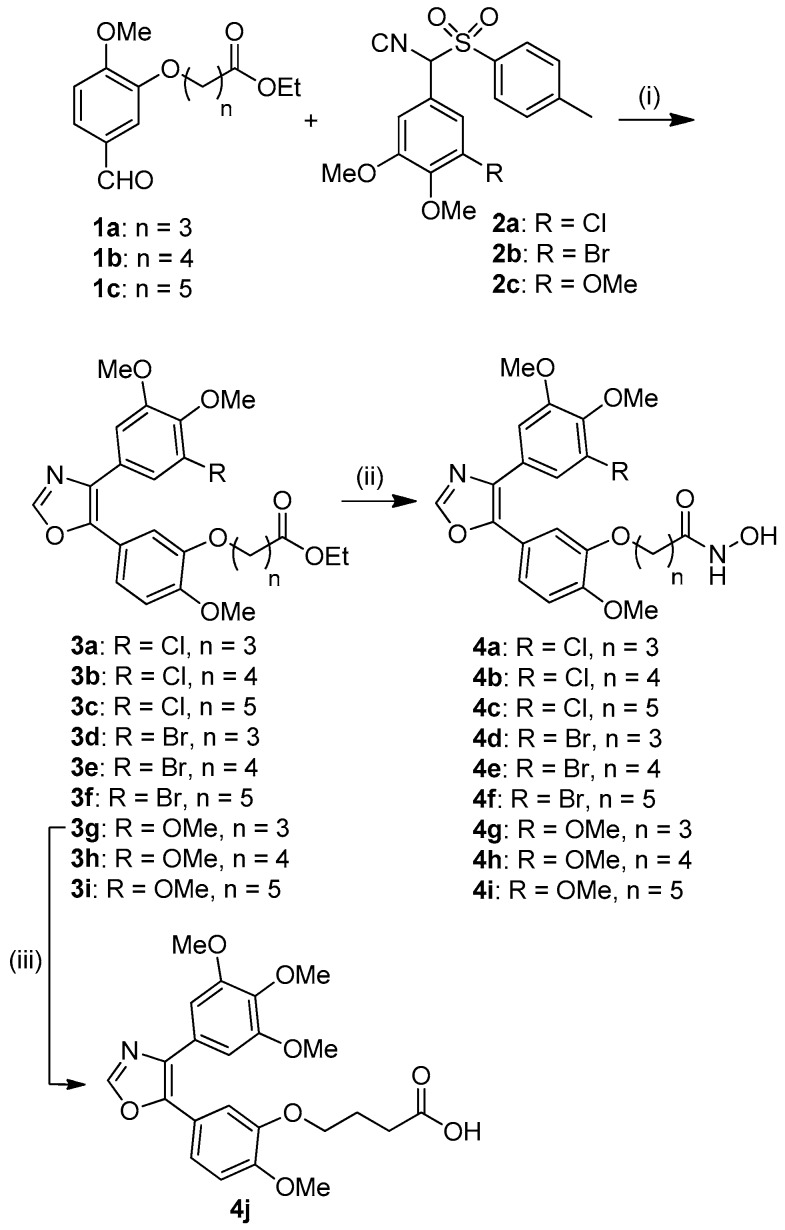
Reagents and conditions: (**i**) K_2_CO_3_, EtOH, reflux, 2 h, 40–53%; (**ii**) 50% H_2_N-OH in H_2_O, NaOH, CH_2_Cl_2_/MeOH (1:2), r.t., 1 h, 64–93%; (**iii**) 1 M aq. NaOH, MeOH, r.t., 24 h, 68%.

The 4,5-diaryloxazoles were synthesized via a Van Leusen reaction. The required starting benzaldehydes **1a**–**c** and TosMIC reagents **2a**–**c** were prepared according to literature procedures, i.e., the former via alkylation of isovanillin with the corresponding ethyl ω-bromoalkanoates, and the latter via dehydration of their tosylmethyl formamide precursors (obtained from reaction of 3-bromo/chloro-4,5-dimethoxybenzaldehyde or 3,4,5-trimethoxybenzaldehyde with toluenesulfinic acid and formamide) [25,26,27,28]. The synthesis of the target hydroxamic acids **4a**–**i** was carried out in two steps. Van Leusen reaction of **1a**–**c** and **2a**–**c** gave the oxazoles **3a**–**i** in moderate yields (Scheme 1). Conversion of the ethyl esters **3a**–**i** to the analogous hydroxamic acids **4a**–**i** was accomplished in moderate to high yields. The target compounds **4a**–**i** were obtained as colorless solids. In addition, carboxylic acid analog **4j** was prepared for comparison purposes and obtained from hydrolysis of **3g** under basic conditions (Scheme 1).

### 2.2. Biological Evaluation 

First, all compounds (**4a**–**j**) were tested for their growth inhibitory potential in cancer cell lines. The new derivatives **4a**–**i**, the new carboxylic acid analog **4j** and its ethyl-ester **3g** were screened in MTT assays for anti-proliferative activity against a panel of six cancer cell lines of four entities as well as against the human endothelial hybrid cell line Ea.Hy926 (Table 1). The bromo derivatives **4d**–**f** were also tested against the non-malignant human dermal fibroblasts HDFa. IC_50_ values of the known HDACi SAHA and the VDA CA-4 were taken from earlier studies for comparison. Compounds **4a**–**i** led to dose-dependent growth inhibition of all cancer cell lines and the endothelial hybrid cells Ea.Hy926. Carboxylic acid **4j** and its ester **3g** did not affect the viability of 518A2 melanoma and HT-29 colon carcinoma cells even at concentrations as high as 50 µM, which suggests that the hydroxamate side chain is crucial for the anti-proliferative activity. 

On average, the CA-4 resistant HT-29 colon carcinoma and the multi-drug resistant MCF-7^Topo^ mamma carcinoma cells were least sensitive to **4a**–**i** [29,30]. In contrast, **4a**–**i** were most active against 518A2 melanoma, HCT-116 colon carcinoma, and endothelial hybrid cells Ea.Hy926. The low IC_50_ values of the compounds **4a**–**i** against Ea.Hy926 cells are indicative of a potential application as a vascular disruptive agent like the parent CA-4. Moreover, the bromo substituted derivatives **4d**–**f** showed a distinct selectivity for cancer and endothelial cells over non-malignant human dermal fibroblasts HDFa. The chloro substituted compounds **4a**–**c** and the bromo substituted compounds **4d**–**f** showed an interesting structure–activity relationship (SAR). The anti-proliferative activity increased with decreasing linker length when going from caproic acid derivatives **4c** and **4f** over valeric acid derivatives **4b** and **4e** to butyric acid derivatives **4a** and **4d**. Interestingly, the trimethoxy derivatives **4g**–**i** did not fit in this SAR since **4i** was on average more cytotoxic than **4h**. Several earlier studies had shown that the substitution of an *m*-methoxy group at the A-ring of CA-4 derivatives by halide increases their activity. We now observed a similar phenomenon since the chloro substituted derivatives **4a**–**c** and the bromo substituted analogs **4d**–**f** were superior to the trimethoxy derivatives **4g**–**i**. Additionally, we determined the IC_50_ values of the test compounds when applied to 518A2 melanoma cells for different incubation periods (24 and 72 h). In the case of the most strongly anti-proliferative compounds (**4a**, **4d**, and **4g**), the IC_50_ values after 72 h were about eight-fold lower compared with those after 24 h. Since most of the investigated cell lines have division periods of 20–30 h, we assume that the test compounds exert their effect by blocking the cell division and by triggering apoptosis [31,32]. Earlier publications by our group disclosed a significant tumor selectivity of the control compounds SAHA and CA-4 (i.e., low activity against CHF/chicken heart fibroblast cells) which can explain the observed tumor selectivity of compounds **4d**–**f** [33,34].

**Figure 2:**
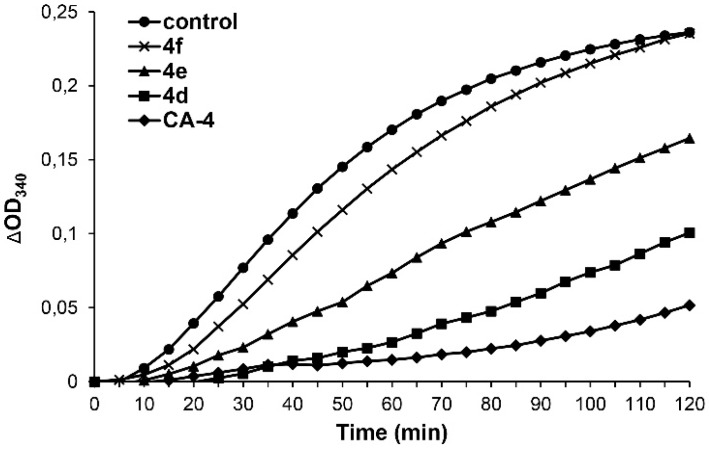
Effects of compounds **4d–f** and CA-4 (10 µM) on the polymerization of tubulin as determined by a turbidimetric cell-free tubulin assay. Data are representative of three independent experiments. ΔOD_340_ is the change in the absorption at 340 nm wavelength with OD_340_ set as 0 at 0 min (*p* ≤ 0.001 for CA-4 and **4d** compared to controls, one-tailed Dunnett post-hoc test).

Next, we investigated how the length of the linker of the test compounds may influence cytoskeletal components, the inhibition of HDAC1 and HDAC6, and cell cycle progression. These analyses were performed only with the bromo substituted derivatives **4d**–**f** which showed slightly higher anti-proliferative activity on average compared to their chloro or methoxy congeners. At first, the new hybrid compounds **4d**–**f** were tested for their potential inhibition of the microtubule assembly, which is a typical feature of the parent compound CA-4. MTT assays already revealed some CA-4 characteristics such as selectivity for EA.Hy926 endothelial hybrid cells and a reduced efficacy against HT-29 colon carcinoma cells [33]. The effect of the test compounds on the polymerization of tubulin was determined in vitro using purified pig brain tubulin (Figure 2). An amount of 10 µM of **4d** inhibited the polymerization of pig brain tubulin nearly completely, while **4e** exhibited a merely moderate inhibitory effect and **4f** virtually none. 

**Figure 3:**
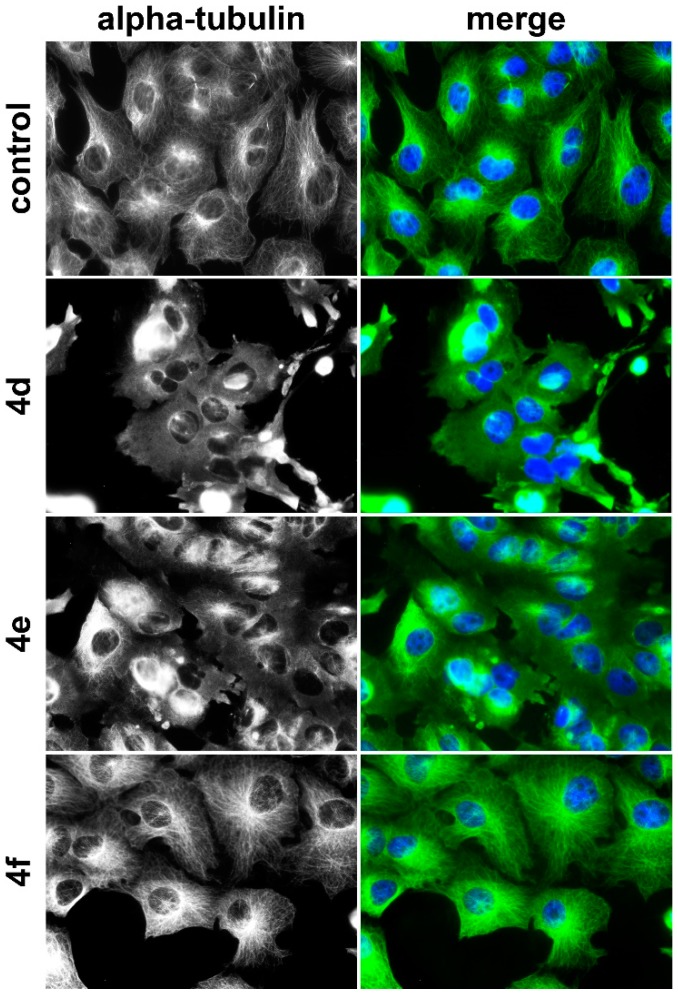
Effect of compounds **4d** (0.5 µM), **4e** (1.5 µM), **4f** (4 µM), and vehicle (DMSO) on the organization of microtubule cytoskeleton in 518A2 melanoma cells after 24 h incubation. Nuclei were counterstained with DAPI (merge, blue); microtubule (green). Pictures are representative of two independent experiments (400× magnification).

These results are in line with the anti-proliferative activity pattern of the compounds and were additionally confirmed on a cellular level by immunostaining of alpha-tubulin in 518A2 melanoma cells (Figure 3). Caproic acid derivative **4f**, showing the highest IC_50_ values in MTT assays, did not affect the microtubule cytoskeleton even at concentrations as high as 4 µM. In contrast, **4e** eroded the highly organized microtubule network, but left some intact clusters especially around the nuclei whereas 0.5 µM of **4d** was enough to cause a complete disruption of the microtubule cytoskeleton. Similar alterations of the cytoskeleton of endothelial Ea.Hy926 cells were observed upon treatment with 0.2 µM of **4d** for 24 h (Figure S1, Supporting Information). The deacetylation of tubulin by compounds **4d** and **4f** is presented below. In addition, elevated levels of reactive oxygen species (ROS, Figure S2, Supporting Information), which are known to trigger apoptosis and reverse chemoresistance in tumors, were observed in 518A2 melanoma cells (**4d**: 241% ± 17; **4e**: 230 ± 31; **4f**: 198 ± 24). Again, as already observed for anti-proliferative activity the ability to elevate ROS levels decreases with increasing linker length.

**Figure 4:**
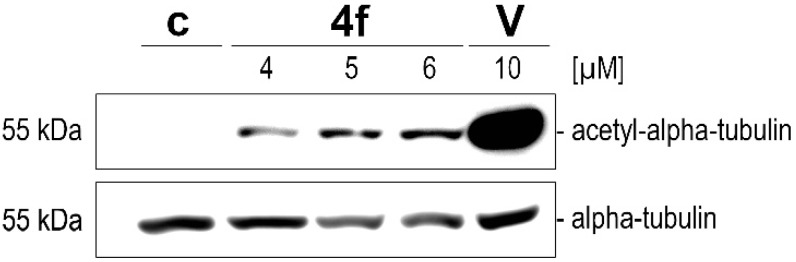
Compound-induced effect on the acetylation of microtubules (acetyl–alpha-tubulin) in 518A2 melanoma cells upon treatment with **4f** (4, 5, and 6 µM), SAHA (10 µM), and vehicle (DMSO) for 24 h. Cells were lysed and the levels of the acetyl–alpha-tubulin was monitored by immunoblotting with the specific antibody. c: control; V: SAHA/vorinostat.

We also investigated the bromo derivatives **4d**–**f** with different linker lengths for their inhibitory effect on the deacetylation capacity of recombinant human HDAC1 and HDAC6 (Table 2). Contrary to the inhibition of tubulin polymerization and cell proliferation, which decreased with growing linker length, the HDAC inhibition increased with linker length. Compound **4d**, the most cytotoxic compound in this row featuring a four-atom spacer, showed only moderate HDAC6 inhibition (IC_50_: 13.8 ± 0.2 µM). Compound **4e**, carrying a five-atom linker, had a distinctly lower IC_50_ value (3.5 ± 0.1 µM), whereas **4f**, the compound with a six-atom linker, had the lowest IC_50_ value of this triad (0.32 ± 0.02 µM), which was even slightly lower than that of the known HDAC6 selective inhibitor tubacin (0.38 ± 0.03 µM). Concerning HDAC1 inhibition, **4d** and **4e** showed similar IC_50_ values (4.0 ± 0.1 and 3.8 ± 0.1 µM) whereas **4f** was again the most potent compound (0.49 ± 0.05 µM). Unlike HDAC1 which is found in the nucleus of cells where it is responsible for the eponymous deacetylation of histones, HDAC6 locates predominantly in the cytoplasm and has several targets including α-tubulin, HSP90, cortactin, and β-catenin [35,36]. The inhibition of HDAC6 induces hyperacetylation of these molecules resulting in a reduction of cell motility, and proliferation, and eventually induces cell death [37]. The ability of compound **4f** to inhibit HDAC6 was confirmed by western blot analyses (Figure 4) as well as by immunofluorescence staining of acetyl–alpha-tubulin in 518A2 melanoma cells (Figures S3 and S4, Supporting Information). In both experiments, treatment of the cells with **4f** caused a distinct increase of acetyl–alpha-tubulin. Thus, a distinct difference between **4d** and **4f** concerning their effects on the microtubule cytoskeleton became visible. While **4d** destroys the microtubules in line with its high tubulin polymerization inhibitory activity, **4f** did not destroy the microtubules yet enhanced the acetylation grade of microtubules due to its strong HDAC6 inhibition. 

**Figure 5:**
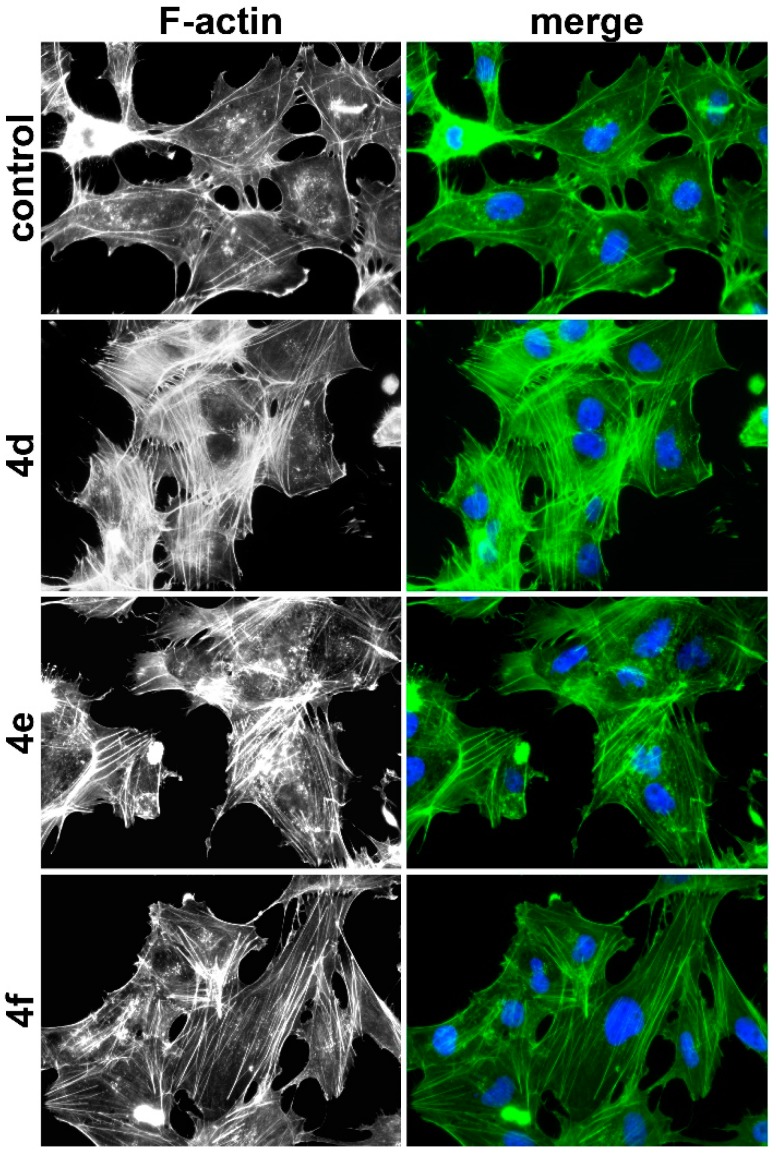
Effect of **4d** (0.5 µM), **4e** (1.5 µM), **4f** (4 µM), and vehicle (DMSO) on the organization of the actin cytoskeleton in 518A2 melanoma cells after 24 h exposure. Fluorescence labeling of filamentous actin (F-actin; green). Nuclei were counterstained with DAPI (merge, blue). Pictures are representative of two independent experiments (400× magnification).

Imidazole-bridged CA-4 derivatives carrying hydroxamic acid appendages had previously been found to induce alterations of the actin cytoskeleton, such as augmented formation of stress fibers to the effect of an impaired cell motility [34,38]. Such alterations are typical reactions to microtubule destabilization and hyperacetylation of cortactin as a consequence of HDAC6 inhibition [39]. Thus, we investigated the bromo derivatives **4d**–**f** for their effect on the actin cytoskeleton of 518A2 melanoma cells (Figure 5). Even though their effects on the microtubules of these cells differed, all of them induced the formation of actin stress fibers which traversed the whole cell body, while the control cells showed only filamentous actin in the periphery. The associated anti-migratory effects of compounds **4d**–**f** were then tested in so-called wound healing assays. In this assay a strip of cells is scratched off a confluent grown cell monolayer of 518A2 cells, followed by monitoring the gap-closing process operating not by proliferation but by active migration (Figures S5 and S6, Supporting Information). The re-closure of the scratch area was significantly retarded in samples treated with compounds **4d**–**f** for 24 h (38–45% wound closure) compared to vehicle treated control cells (63% wound closure).

**Figure 6:**
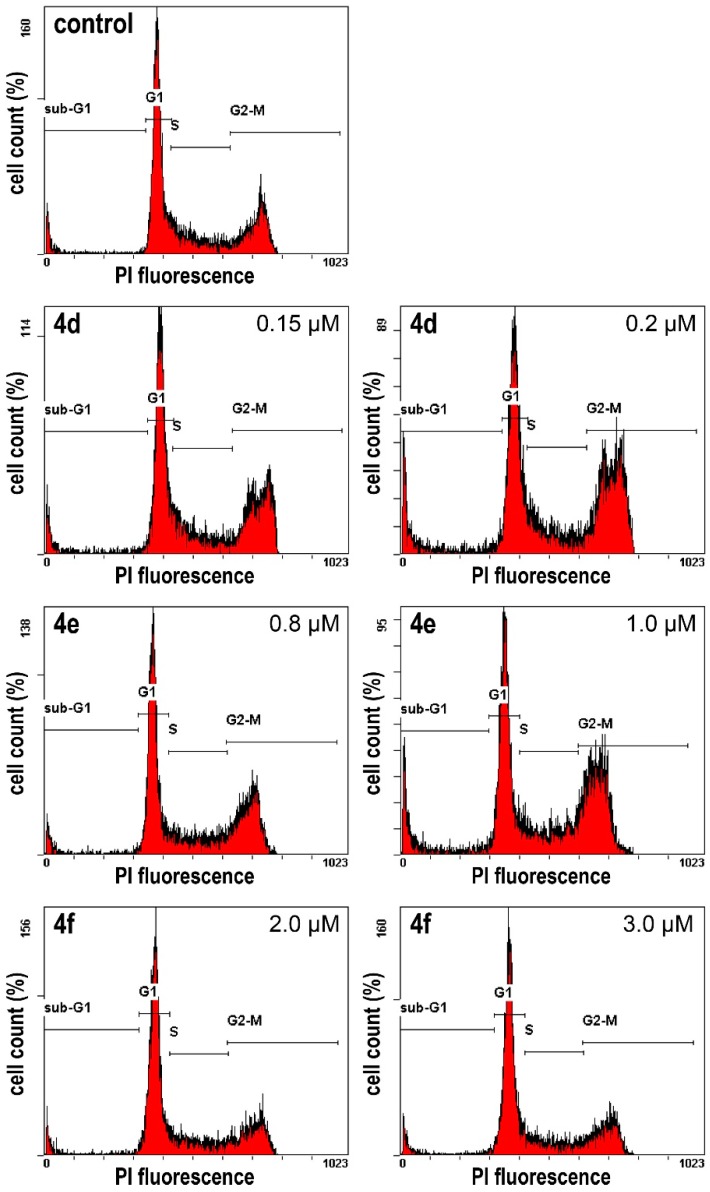
Effect of **4d** (0.15 and 0.2 µM), **4e** (0.8 and 1 µM), **4f** (2 and 3 µM) or control (DMSO) on the proportions of 518A2 melanoma cells in G1, S, and G2/M phase of the cell cycle as well as the percentages of apoptotic cells in sub-G1 as determined by flow cytometry after PI staining. Cells were treated with the test compounds for 24 h. Cell cycle profiles are representatives of at least three independent experiments (*p* ≤ 0.05 for cells in G1-phase treated with **4d–f** compared with those of vehicle-treated controls, two-tailed Games–Howell’s post-hoc test).

Stress fiber formation in combination with microtubule destruction frequently leads to the arrest of cells in the G2/M phase of the cell cycle. By contrast, HDAC inhibition typically induces an arrest of cells in the G1 phase [40,41,42,43,44,45]. To investigate whether microtubule destabilization or HDAC inhibition of the test compounds **4d**–**f** prevails on cell cycle regulation, cell cycle arresting effects were tested in 518A2 melanoma cells by FACS analysis (Figure 6, Table 3). As expected, the strongly microtubule-destabilizing compounds **4d** and **4e** led to an accumulation of 518A2 cells in G2/M phase. In contrast, the stronger HDAC inhibitor **4f**, which lacks microtubule destabilizing activity, induced a G1 phase arrest in the investigated melanoma cells.

**Figure 7:**
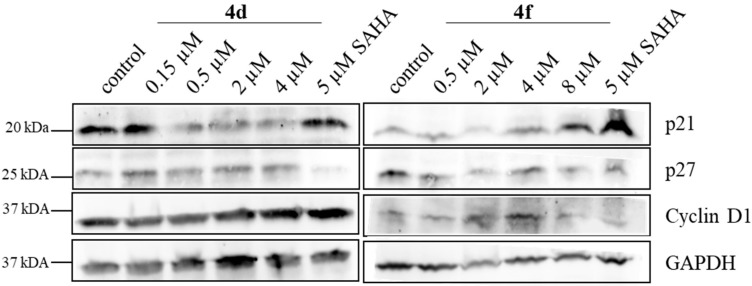
Compound-induced effects on cell cycle regulatory proteins in 518A2 melanoma cells after 24 h treatment with **4d**, **4f**, and SAHA. GAPDH served as a loading control. Representative data of three independent experiments are shown.

The effects of **4d** on the cell cycle regulatory proteins p21, p27, and cyclin D1 in 518A2 melanoma cells were investigated (Figure 7). At higher doses **4d** increased the level of cyclin D1 which was comparable with the effect on cyclin D1 by SAHA. It seems that the HDAC inhibitory properties of **4d** at higher concentrations caused the induction of cyclin D1. In addition, **4d** distinctly suppressed p21 expression while SAHA showed no effects on p21. It is conceivable that the observed p21 suppression is associated with the cytoskeleton targeting of **4d** which is also in line with the G2/M arrest caused by **4d** in 518A2 cells. In contrast to that, **4f** seemingly upregulated p21 expression in a dose-dependent way in congruence to SAHA. 

Finally, compound **4d** was tested concerning in vivo applicability and the toxicity of compound **4d** to mice was investigated (Figure S7, Supporting Information). High doses of **4d** (1 × 100 mg/kg i.p., 1 × 200 mg/kg orally) were tolerated well by the treated mice and they showed no signs of toxicity (i.e., no weight loss, weight changes of maximal 5%, normal behavior). Thus, a more thorough in vivo investigation of **4d** in suitable tumor xenograft models is recommended due to the manageable toxicity profile of this compound.

### 2.3. In Silico Evaluation

Structure-based docking was used to explore the interactions of the different compounds with the respective target proteins on a molecular level. Molecular docking is an efficient technique for calculating the binding modes of a compound and estimating their binding affinities. Docking in general is a selection and optimization process, trying to find the best fit of a molecule in the binding site of a protein according to a scoring function [46]. 

To evaluate the role of the varying linker-length, the CA-4 derivative (Cap) and the hydroxamic acid group (ZBG) for the binding affinity to tubulin, docking studies were performed for the structures **4d**–**f**. In the tubulin structure 5LYJ, used for the docking studies, the microtubule-destabilizing agent CA-4 binds to the colchicine site of the tubulin β-chain close to the interface of the neighboring tubulin α-chain, which restrains this binding site. The CA-4 site is a buried hydrophobic pocket shaped by residues Val238, Cys241, Leu242, Leu248, Ala250, Leu255, Ala316, Ile318, Ala354, and Ile378 [47]. 

**Figure 8:**
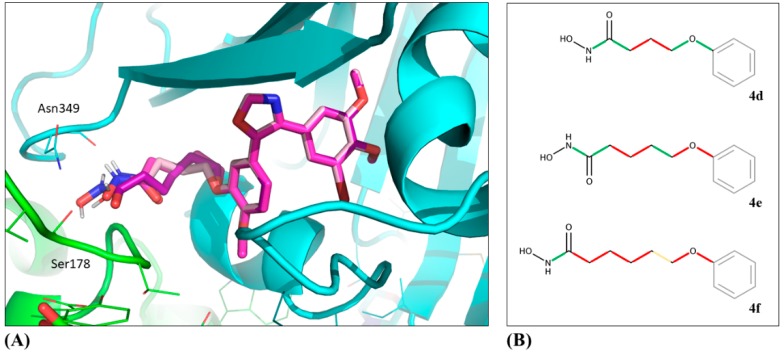
(**A**) Superposition of selected binding poses for **4d** (light-pink), **4e** (medium-pink) and **4f** (dark-pink) in tubulin structure 5LYJ, exported from SeeSAR and visualized with PyMOL. (**B**) Illustration of frequent (green), occasional (yellow), and rare (red) torsions within the linker and hydroxamic acid group.

The docking studies for tubulin showed almost identical binding positions of the CA-4 cap of the molecules **4d**–**f** in the hydrophobic colchicine binding pocket similar to the original ligand CA-4 (Figure 8A). The linker extended towards the tubulin α-chain cap and the hydroxamic acid was predicted to form hydrogen bonds with Asn349 from the β-chain (**4d**–**f**) and potentially with Ser178 from the α–chain (**4f**). As summarized in Table 4, the estimated affinities slightly decreased with increasing linker length, a finding that is in agreement with the wet-lab results. Another observation was that the longer the linker, the more twisted it needed to be to fit into the capped cavity. Thus, the chain had to adopt torsions which are only seldom observed in crystal structures and which are considered as unfavorable; see Figure 8B [48,49]. 

**Figure 9:**
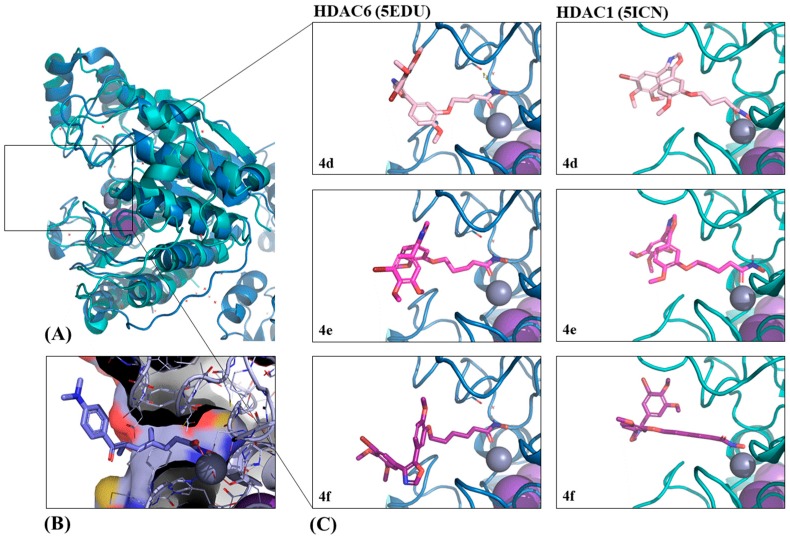
(**A**) Aligned PDB structures of HDAC1 (PDB code: 5ICN) and HDAC6 (PDB code: 5EDU). (**B**) Surface visualization of HDAC6 binding site with co-crystallized ligand trichostatin A (PDB code: 5EDU). (**C**) Selected binding pose predictions exported from SeeSAR and visualized with PyMOL.

To explore reasons for the differences in the HDAC inhibitory potency of the compounds on a molecular level, computational docking studies were performed. The calculations were based on the X-ray structures 5ICN (HDAC1) and 5EDU (HDAC6) and were carried out for the compounds **4d**–**f** as well as for vorinostat and the respective co-crystallized ligands (see methods section). Generally, HDACs feature an active site with a relatively narrow tunnel pointing towards the buried catalytic zinc(II) cation to which the hydroxamic acid of the natural substrates and inhibitors such as vorinostat binds. The two HDAC structures used in this study exhibit very similar binding sites and can be superimposed with a low backbone RMSD of 1.9Å (superposition calculated with PyMol, see Figure 9A).

The best poses calculated with SeeSAR for all compounds (including re-docking of the co-crystallized ligands and vorinostat) showed a similar orientation of the linker threading through the narrow tunnel and the hydroxamic acid chelating the zinc(II) cation (Figure 9B). While the estimated affinities are all in a similar range, the values suggest that a longer linker is more favorable (Table 5 and Table 6), which is in accordance with the experimental results (Table 2). This could be due to the better fit through the long (e.g., ~10 Å in HDAC6) and narrow active site tunnel, which would allow the hydroxamic acid group with longer linker length to reach the zinc ion more easily [50]. In contrast, the distance between the hydroxamic acid terminus and the first benzene attached to the linker in **4d** is only ~8 Å, which might make it difficult to find a good hydroxamic acid fit without causing a clash of the bulky CA-4 derived capping group with the protein.

In all docked compounds, the hydroxamic acid group formed hydrogen bonds with the buried amino acids His610 and Gly619 of the HDAC6 structure 5EDU (co-crystallized to trichostatin A) as well as a bond to the zinc ion [50]. Generally, the hydroxamic acid and the more buried part of the linker of all three compounds **4d**–**f** overlap also with the respective part of the co-crystallized trichostatin A (Figure 9C, left panel). The capping group, which is rather solvent exposed, was more variable in its position, but may form hydrogen bonds with His651 and Phe680. 

The docking results for HDAC1 structure 5ICN were more diverse. This could be due to the fact that the structure is co-crystallized with a peptide inhibitor (H4K16Hx) and the loop around residue Asp99 undergoes a significant rearrangement to allow binding of the peptide [51]. Note that there are only two HDAC1 structures available to date (5ICN and 4BKX), of which 4BKX is an apo structure, so 5ICN was the only available complex structure. Reasonable poses for all compounds could be generated with LeadIT, whereas the optimization in SeeSAR was successful for all compounds but **4d** (Figure 9C, right panel)**.** Furthermore, the binding poses of the three compounds **4d**–**f** and vorinostat differed more in the hydrogen and metal bonds formed by the hydroxamic acid. While the hydroxamic acid group of **4f** was predicted to form hydrogen bonds with Tyr303 and His141, it was predicted to only form one hydrogen bond with Gly301 in **4e.** The capping group might form H-Bonds with Asn95 and Gly149.

While the results were more distinct for HDAC6 (where a good protein–ligand complex structure was available), generally, the docking analysis showed that the molecules with longer linker length tend to have a higher affinity to HDAC1 and HDAC6. 

## 3. Discussion

Recently, we presented conjugates of imidazoles and hydroxamic acids which combine HDAC inhibition with cytoskeletal modulation [34,38,52]. However, conjugates with imidazoles derived from CA-4 had lost crucial CA-4 typical properties such as inhibition of the polymerization of tubulin. Herein, we introduced a new series of conjugates of oxazole-bridged CA-4 and hydroxamic acids that show dual tubulin and HDAC interference which mainly depends on the length of the linker connecting these two fragments. In terms of cytotoxicity, inhibition of tubulin polymerization, and generation of ROS, the potency of these conjugates grows with decreasing linker length. The strong anti-proliferative effect especially of the derivatives with the shortest linkers **4a**, **4d**, and **4g** is probably due to their interference with formation of the spindle apparatus during mitosis. The derivatives with longer tethers increasingly lose this property resulting in a distinctly reduced anti-proliferative effect which was confirmed by in silico studies and was attributed to the increasingly tensed fitting of the linkers in the capped cavity. 

By destabilizing the microtubules, the new conjugates affect the cell division and probably the integrity of endothelial cell layers. Moreover, they showed a distinct specificity for endothelial hybrid cells Ea.Hy926 over non-malignant cells HDFa. Both properties underline the great potential of these derivatives as VDAs which trigger the collapse of the leaky and low-quality blood vessels of tumors by destabilizing their endothelial lining, initiating necrosis in the core of the tumor [19]. An assessment of the vascular-disrupting activity of the new compounds is currently underway. 

The HDAC inhibitory effect of the new conjugates showed the opposite dependency on linker length. The IC_50_ values for inhibition of both HDAC1 and HDAC6 decreased with increasing linker length reflecting the fact that the active site of the zinc-dependent HDACs is accessible for HDACi only by a long narrow hydrophobic channel. We rationalized this trend by in silico experiments. 

The observed G1-arrest of 518A2 cells by **4f** matches with similar observations for other HDACi. The MDA-typical G2/M arrest by derivatives **4d** and **4e** is in line with their pronounced microtubule disrupting effect which probably overrides their HDACi activity. The stress fiber induction as well as the anti-migratory activity observed for **4d**–**f** was independent of linker length. Together with the lack of toxicity in mice, compound **4d** appears to be an especially promising drug candidate.

In this work we were able to demonstrate the anti-proliferative, cell-cycle arresting, microtubule-destabilizing and actin stress fiber-inducing effects of novel CA-4 analogues with the potential to inhibit histone deacetylases. The results are indicative of the great potential of this new class of compounds which probably affect the tumor vasculature either by inhibiting angiogenesis or by disruption of already established tumor blood vessels. Investigations of these anti-vascular effects as well as their in vivo activity are currently underway.

## 4. Materials and Methods 

### 4.1. General Procedures

The following instruments were used: melting points (uncorrected), Gallenkamp; IR spectra, Perkin-Elmer Spectrum One FT-IR spectrophotometer with ATR sampling unit; nuclear magnetic resonance spectra, BRUKER Avance 300 spectrometer; chemical shifts are given in parts per million (δ) downfield from tetramethylsilane as internal standard; mass spectra, Varian MAT 311A (EI) or UPLC/Orbitrap MS system (ESI); microanalyses, Perkin-Elmer 2400 CHN elemental analyzer. All tested compounds were >95% pure by elemental analysis.

### 4.2. Materials

The known starting compounds **1a** and **2a**–**c** were prepared according to literature procedures [25,26,27]. The new compounds **1b** and **1c** were synthesized analogously to **1a** (see below). All other starting compounds and reagents were purchased from Sigma–Aldrich.

### 4.3. General Procedure for the Synthesis of Intermediates 1b and 1c

Isovanillin (152 mg, 1.0 mmol) was dissolved in MeCN (5 mL) and cesium carbonate (652 mg, 2.0 mmol) was added. After stirring at 90 °C for 0.5 h, ethyl 5-bromovalerate (328 µL, 2.0 mmol) or ethyl 6-bromohexanoate (356 µL, 2.0 mmol), respectively, was added and the reaction mixture was stirred at 90 °C for 2 h. After filtration through celite, the solvent was removed under reduced pressure and the residue was washed twice with *n*-hexane. The resulting oil was dried in vacuum and used for the next step without further purification.

**Ethyl 5-(1-methoxy-4-formyl-2-phenoxy)valerate (1b).** Yield: 130 mg (0.46 mmol, 46%); colorless oil. *υ*_max_(ATR)/cm^−1^ 2941, 2881, 2841, 1728, 1683, 1585, 1509, 1462, 1436, 1393, 1374, 1340, 1262, 1238, 1159, 1133, 1096, 1019, 935, 864, 809, 780, 749, 737, 641. ^1^H NMR (300 MHz, CDCl_3_): *δ* = 1.23 (t, 3H, *J* = 7.1 Hz), 1.8–2.0 (m, 4H), 2.38 (t, 2H, *J* = 7.3 Hz), 3.93 (s, 3H), 4.0–4.2 (m, 4H), 6.95 (d, 1H, *J* = 8.2 Hz), 7.37 (s, 1H), 7.44 (d, 2H, *J* = 8.2 Hz), 9.82 ppm (s, 1H). ^13^C NMR (75.5 MHz, CDCl_3_): *δ* = 14.2, 21.6, 28.5, 33.9, 56.2, 60.3, 68.5, 110.5, 110.7, 126.7, 130.1, 149.0, 154.9, 173.3, 190.9 ppm. *m/z* (%) 280 (14) [M^+^], 235 (24), 151 (57), 129 (98), 101 (100), 83 (82), 55 (56), 43 (15).

**Ethyl 6-(1-methoxy-4-formyl-2-phenoxy)hexanoate (1c).** Yield: 110 mg (0.37 mmol, 37%); colorless oil. *υ*_max_(ATR)/cm^−1^ 2940, 2870, 1729, 1685, 1585, 1510, 1462, 1436, 1394, 1374, 1341, 1264, 1239, 1161, 1134, 1068, 1021, 862, 811, 779, 749, 729, 641. ^1^H NMR (300 MHz, CDCl_3_): *δ* = 1.23 (t, 3H, *J* = 7.1 Hz), 1.4–1.6 (m, 2H), 1.6–1.8 (m, 2H), 1.8–1.9 (m, 2H), 2.32 (t, 2H, *J* = 7.3 Hz), 4.01 (s, 3H), 4.0–4.2 (m, 4H), 6.95 (d, 1H, *J* = 8.2 Hz), 7.37 (s, 1H), 7.44 (d, 2H, *J* = 8.2 Hz), 9.82 ppm (s, 1H). ^13^C NMR (75.5 MHz, CDCl_3_): *δ* = 14.2, 24.7, 25.6, 28.7, 34.2, 56.2, 60.2, 68.8, 110.4, 110.6, 118.0, 126.6, 130.1, 149.1, 154.9, 173.6, 190.9 ppm. *m/z* (%) 294 (48) [M^+^], 249 (35), 152 (98), 143 (100), 115 (54), 97 (97), 69 (86), 55 (22), 41 (28).

### 4.4. General Procedure for the Synthesis of Intermediates **3a**–**i**

The respective compounds **1** (0.42 mmol) and **2** (0.42 mmol) and K_2_CO_3_ (590 mg, 4.3 mmol) were suspended in ethanol and stirred under reflux for 2 h. The solvent was removed and the residue dissolved in ethyl acetate and washed with water. The organic phase was dried over Na_2_SO_4_, concentrated in vacuum, and the residue was purified by column chromatography (silica gel 60).

**Ethyl 4-[1-methoxy-4-(4’-(3’’-chloro-4’’,5’’-dimethoxyphenyl)oxazol-5’-yl)-2-phenoxy] butyrate (3a).** Compound **3a** was obtained from **1a** (113 mg, 0.42 mmol), **2a** (155 mg, 0.42 mmol) and K_2_CO_3_ (590 mg, 4.3 mmol). Yield: 103 mg (0.22 mmol, 52%); R*f* = 0.39 (ethyl acetate/*n*-hexane, 2:3); colorless oil. *υ*_max_(ATR)/cm^−1^ 2969, 2938, 2876, 2837, 1731, 1683, 1629, 1561, 1513, 1489, 1463, 1443, 1414, 1399, 1366, 1303, 1254, 1229, 1174, 1140, 1117, 1105, 1047, 1024, 1000, 960, 938, 892, 853, 810, 774, 758, 734, 709, 659, 629. ^1^H NMR (300 MHz, CDCl_3_): *δ* = 1.23 (t, 3H, *J* = 7.1 Hz), 2.0–2.2 (m, 2H), 2.49 (t, 2H, *J* = 7.3 Hz), 3.81 (s, 3H), 3.87 (s, 3H), 3.88 (s, 3H), 3.98 (t, 2H, *J* = 6.3 Hz), 4.11 (q, 2H, *J* = 7.2 Hz), 6.87 (d, 1H, *J* = 8.4 Hz), 7.1–7.2 (m, 3H), 7.29 (s, 1H), 7.88 ppm (s, 1H). ^13^C NMR (75.5 MHz, CDCl_3_): *δ* = 14.2, 24.5, 30.7, 56.0, 56.1, 60.4, 60.8, 68.1, 110.6, 111.7, 112.0, 120.4, 120.9, 121.1, 128.3, 128.7, 132.3, 146.1, 148.4, 149.3, 150.4, 153.8, 173.0 ppm. *m/z* (%) 477 (61) [M^+^], 475 (100) [M^+^], 432 (11), 430 (31), 361 (8), 265 (7), 115 (94), 87 (67), 43 (11).

**Ethyl 5-[1-methoxy-4-(4’-(3’’-chloro-4’’,5’’-dimethoxyphenyl)oxazol-5’-yl)-2-phenoxy]valerate (3b).** Compound **3b** was obtained from **1b** (126 mg, 0.45 mmol), **2a** (165 mg, 0.45 mmol) and K_2_CO_3_ (590 mg, 4.3 mmol). Yield: 98 mg (0.20 mmol, 44%); R*f* = 0.42 (ethyl acetate/*n*-hexane, 2:3); colorless oil. *υ*_max_(ATR)/cm^−1^ 2938, 2870, 2838, 1730, 1603, 1591, 1564, 1513, 1489, 1463, 1414, 1365, 1324, 1294, 1254, 1229, 1203, 1164, 1140, 1105, 1048, 1024, 938, 854, 811, 776, 758, 710, 659, 628. ^1^H NMR (300 MHz, CDCl_3_): *δ* = 1.23 (t, 3H, *J* = 7.1 Hz), 1.7–1.9 (m, 2H), 2.35 (t, 2H, *J* = 7.3 Hz), 3.81 (s, 3H), 3.87 (s, 3H), 3.88 (s, 3H), 3.93 (t, 2H, *J* = 6.1 Hz), 4.10 (q, 2H, *J* = 7.1 Hz), 6.87 (d, 1H, *J* = 8.4 Hz), 7.10 (s, 1H), 7.1–7.2 (m, 2H), 7.30 (s, 1H), 7.88 ppm (s, 1H). ^13^C NMR (75.5 MHz, CDCl_3_): *δ* = 14.2, 21.6, 28.6, 33.9, 56.0, 56.1, 60.3, 60.8, 68.7, 110.6, 111.7, 120.2, 120.9, 121.1, 128.3, 128.7, 132.3, 145.2, 146.1, 148.5, 149.3, 150.3, 153.8, 173.4 ppm. *m/z* (%) 491 (43) [M^+^], 489 (100) [M^+^], 446 (13), 444 (41), 361 (31), 346 (17), 168 (14), 129 (98), 101 (98), 83 (56), 55 (39), 43 (11).

**Ethyl 6-[1-methoxy-4-(4’-(3’’-chloro-4’’,5’’-dimethoxyphenyl)oxazol-5’-yl)-2-phenoxy] hexanoate (3c).** Compound **3c** was obtained from **1c** (91 mg, 0.31 mmol), **2a** (113 mg, 0.31 mmol) and K_2_CO_3_ (590 mg, 4.3 mmol). Yield: 63 mg (0.13 mmol, 42%); R*f* = 0.45 (ethyl acetate/*n*-hexane, 2:3); colorless oil. *υ*_max_(ATR)/cm^−1^ 2939, 2871, 2837, 1730, 1605, 1591, 1563, 1513, 1489, 1463, 1414, 1366, 1325, 1300, 1255, 1229, 1203, 1175, 1165, 1140, 1105, 1048, 1024, 1001, 939, 854, 821, 775, 758, 710, 659, 629. ^1^H NMR (300 MHz, CDCl_3_): *δ* = 1.23 (t, 3H, *J* = 7.1 Hz), 1.4-1.5 (m, 2H), 1.6–1.7 (m, 2H), 1.8–1.9 (m, 2H), 2.30 (t, 2H, *J* = 7.3 Hz), 3.80 (s, 3H), 3.87 (s, 3H), 3.88 (s, 3H), 3.92 (t, 2H, *J* = 6.7 Hz), 4.10 (q, 2H, *J* = 7.1 Hz), 6.88 (d, 1H, *J* = 8.4 Hz), 7.10 (s, 1H), 7.1-7.2 (m, 2H), 7.30 (s, 1H), 7.89 ppm (s, 1H). ^13^C NMR (75.5 MHz, CDCl_3_): *δ* = 14.2, 24.7, 25.6, 28.8, 34.2, 56.0, 56.1, 60.2, 60.8, 68.9, 110.6, 111.6, 120.1, 120.9, 121.1, 128.3, 128.7, 132.3, 145.2, 146.1, 148.6, 149.3, 150.3, 153.8, 173.6 ppm. *m/z* (%) 505 (68) [M^+^], 503 (100) [M^+^], 458 (11), 361 (37), 346 (13), 143 (51), 115 (16), 97 (28), 69 (26).

**Ethyl 4-[1-methoxy-4-(4’-(3’’-bromo-4’’,5’’-dimethoxyphenyl)oxazol-5’-yl)-2-phenoxy] butyrate (3d).** Compound **3d** was obtained from **1a** (125 mg, 0.47 mmol), **2b** (192 mg, 0.47 mmol) and K_2_CO_3_ (590 mg, 4.3 mmol). Yield: 100 mg (0.19 mmol, 41%); R*f* = 0.25 (ethyl acetate/*n*-hexane, 1:2); colorless oil. *υ*_max_(ATR)/cm^−1^ 3118, 2986, 2940, 2837, 1739, 1592, 1561, 1514, 1488, 1468, 1441, 1404, 1388, 1362, 1349, 1267, 1256, 1241, 1226, 1208, 1179, 1145, 1124, 1108, 1072, 1048, 1025, 997, 960, 944, 859, 846, 820, 807, 773, 754, 699, 654. ^1^H NMR (300 MHz, CDCl_3_): *δ* = 1.23 (t, 3H, *J* = 7.2 Hz), 2.0–2.2 (m, 2H), 2.50 (t, 2H, *J* = 7.3 Hz), 3.80 (s, 3H), 3.86 (s, 3H), 3.88 (s, 3H), 3.98 (t, 2H, *J* = 6.3 Hz), 4.11 (q, 2H, *J* = 7.2 Hz), 6.88 (d, 1H, *J* = 8.4 Hz), 7.12 (s, 1H), 7.1–7.2 (m, 2H), 7.46 (s, 1H), 7.88 ppm (s, 1H). ^13^C NMR (75.5 MHz, CDCl_3_): *δ* = 14.2, 24.5, 30.8, 56.0, 56.1, 60.4, 60.7, 68.2, 111.4, 111.7, 111.9, 117.6, 120.3, 120.9, 123.9, 129.3, 146.1, 148.4, 149.3, 150.4, 153.7, 173.0 ppm. *m/z* (%) 521 (38) [M^+^], 519 (37) [M^+^], 476 (6), 474 (5), 115 (100), 87 (79), 43 (14).

**Ethyl 5-[1-methoxy-4-(4’-(3’’-bromo-4’’,5’’-dimethoxyphenyl)oxazol-5’-yl)-2-phenoxy]valerate (3e).** Compound **3e** was obtained from **1b** (112 mg, 0.40 mmol) and **2b** (164 mg, 0.40 mmol) and K_2_CO_3_ (590 mg, 4.3 mmol). Yield: 110 mg (0.21 mmol, 53%); R*f* = 0.33 (ethyl acetate/*n*-hexane, 2:3); colorless oil. *υ*_max_(ATR)/cm^−1^ 3128, 2938, 2838, 1730, 1591, 1557, 1513, 1485, 1463, 1414, 1364, 1254, 1230, 1201, 1174, 1162, 1140, 1105, 1043, 1025, 1000, 939, 897, 854, 807, 776, 754, 698, 659, 628. ^1^H NMR (300 MHz, CDCl_3_): *δ* = 1.23 (t, 3H, *J* = 7.1 Hz), 1.7–1.9 (m, 4H), 2.36 (t, 2H, *J* = 7.3 Hz), 3.80 (s, 3H), 3.86 (s, 3H), 3.88 (s, 3H), 3.93 (t, 2H, *J* = 6.1 Hz), 4.10 (q, 2H, *J* = 7.1 Hz), 6.88 (d, 1H, *J* = 8.4 Hz), 7.11 (s, 1H), 7.1–7.2 (m, 2H), 7.47 (s, 1H), 7.88 ppm (s, 1H). ^13^C NMR (75.5 MHz, CDCl_3_): *δ* = 14.2, 21.6, 28.6, 33.9, 56.0, 56.1, 60.3, 60.7, 68.7, 111.4, 111.6, 111.7, 117.6, 120.1, 120.9, 123.9, 129.4, 132.1, 146.1, 146.3, 148.6, 149.3, 150.3, 153.7, 173.3 ppm. *m/z* (%) 535 (95) [M^+^], 533 (95) [M^+^], 490 (17), 488 (16), 407 (15), 405 (15), 129 (96), 101 (100), 83 (42), 55 (40).

**Ethyl 6-[1-methoxy-4-(4’-(3’’-bromo-4’’,5’’-dimethoxyphenyl)oxazol-5’-yl)-2-phenoxy] hexanoate (3f).** Compound **3f** was obtained from **1c** (113 mg, 0.38 mmol), **2b** (158 mg, 0.38 mmol) and K_2_CO_3_ (590 mg, 4.3 mmol). Yield: 80 mg (0.15 mmol, 40%); R*f* = 0.45 (ethyl acetate/*n*-hexane, 2:3); colorless oil. *υ*_max_(ATR)/cm^−1^ 2940, 2869, 1730, 1590, 1558, 1513, 1485, 1463, 1414, 1364, 1323, 1253, 1229, 1175, 1160, 1140, 1105, 1066, 1043, 1025, 999, 938, 853, 806, 775, 753, 697, 659, 628. ^1^H NMR (300 MHz, CDCl_3_): *δ* = 1.23 (t, 3H, *J* = 7.1 Hz), 1.4–1.5 (m, 2H), 1.6–1.8 (m, 2H), 1.8–1.9 (m, 2H), 2.30 (t, 2H, *J* = 7.3 Hz), 3.80 (s, 3H), 3.86 (s, 3H), 3.88 (s, 3H), 3.9-4.0 (m, 5H), 4.10 (q, 2H, *J* = 7.1 Hz), 6.88 (d, 1H, *J* = 8.4 Hz), 7.10 (s, 1H), 7.1–7.2 (m, 2H), 7.47 (s, 1H), 7.88 ppm (s, 1H). ^13^C NMR (75.5 MHz, CDCl_3_): *δ* = 14.2, 24.7, 25.6, 28.8, 34.2, 56.0, 56.1, 60.2, 60.7, 68.9, 111.4, 111.6, 111.7, 117.6, 120.0, 120.9, 123.9, 129.4, 132.1, 146.2, 148.6, 149.3, 150.3, 153.7, 173.6 ppm. *m/z* (%) 549 (99) [M^+^], 547 (100) [M^+^], 407 (31), 405 (32), 143 (77), 115 (20), 97 (32), 69 (27).

**Ethyl 4-[1-methoxy-4-(4’-(3’’,4’’,5’’-trimethoxyphenyl)oxazol-5’-yl)-2-phenoxy]butyrate (3g).** Compound **3g** was obtained from **1a** (134 mg, 0.50 mmol), **2c** (182 mg, 0.50 mmol) and K_2_CO_3_ (590 mg, 4.3 mmol). Yield: 124 mg (0.26 mmol, 52%); R*f* = 0.31 (ethyl acetate/*n*-hexane, 1:1); colorless oil. *υ*_max_(ATR)/cm^−1^ 2939, 2840, 1730, 1583, 1515, 1463, 1415, 1372, 1306, 1255, 1236, 1173, 1122, 1022, 1004, 956, 938, 885, 837, 811, 768, 733, 659, 629. ^1^H NMR (300 MHz, CDCl_3_): *δ* = 1.27 (t, 3H, *J* = 7.1 Hz), 2.1–2.2 (m, 2H), 2.53 (t, 2H, *J* = 7.3 Hz), 3.81 (s, 6H), 3.90 (s, 3H), 3.91 (s, 3H), 4.02 (t, 2H, *J* = 6.3 Hz), 4.15 (q, 2H, *J* = 7.1 Hz), 6.90 (d, 1H, *J* = 8.4 Hz), 7.12 (s, 1H), 6.94 (s, 2H), 7.17 (s, 1H), 7.25 (d, 1H, *J* = 8.4 Hz), 7.93 ppm (s, 1H). ^13^C NMR (75.5 MHz, CDCl_3_): *δ* = 14.2, 24.5, 30.7, 56.0, 56.1, 60.4, 60.9, 68.0, 104.9, 111.5, 112.1, 120.4, 121.3, 127.6, 133.6, 137.9, 145.6, 148.3, 149.2, 150.1, 153.3, 173.0 ppm. *m/z* (%) 471 (95) [M^+^], 456 (13), 426 (31), 195 (17), 115 (100), 87 (99), 69 (14), 43 (24). Elemental analysis calculated (%) for C_25_H_29_NO_8_: C 63.68, H 6.20, N 2.97. Found: C 63.65, H 6.18, N 2.96.

**Ethyl 5-[1-methoxy-4-(4’-(3’’,4’’,5’’-trimethoxyphenyl)oxazol-5’-yl)-2-phenoxy]valerate (3h).** Compound **3h** was obtained from **1b** (130 mg, 0.46 mmol), **2c** (168 mg, 0.46 mmol) and K_2_CO_3_ (590 mg, 4.3 mmol). Yield: 107 mg (0.22 mmol, 48%); R*f* = 0.35 (ethyl acetate/*n*-hexane, 1:1); colorless oil. *υ*_max_(ATR)/cm^−1^ 2938, 2840, 1730, 1583, 1515, 1455, 1415, 1372, 1330, 1305, 1255, 1237, 1171, 1123, 1022, 1004, 938, 894, 837, 811, 768, 734, 659, 629. ^1^H NMR (300 MHz, CDCl_3_): *δ* = 1.23 (t, 3H, *J* = 7.1 Hz), 1.7–1.9 (m, 4H), 2.34 (d, 2H, *J* = 7.2 Hz), 3.77 (s, 6H), 3.85 (s, 3H), 3.87 (s, 3H), 3.93 (t, 2H, *J* = 6.1 Hz), 4.10 (q, 2H, *J* = 7.1 Hz), 6.86 (d, 1H, *J* = 8.4 Hz), 6.90 (s, 2H), 7.11 (s, 1H), 7.20 (d, 1H, *J* = 8.4 Hz), 7.89 ppm (s, 1H). ^13^C NMR (75.5 MHz, CDCl_3_): *δ* = 14.2, 21.6, 28.6, 33.9, 56.0, 56.1, 60.3, 60.9, 68.6, 105.0, 111.5, 111.9, 120.3, 121.3, 127.6, 133.6, 138.0, 145.7, 148.5, 149.2, 150.1, 153.3, 173.3 ppm. *m/z* (%) 485 (100) [M^+^], 440 (15), 401 (16), 153 (16), 129 (99), 101 (73), 83 (26), 55 (17).

**Ethyl 6-[1-methoxy-4-(4’-(3’’,4’’,5’’-dimethoxyphenyl)oxazol-5’-yl)-2-phenoxy]hexanoate (3i).** Compound **3i** was obtained from **1c** (116 mg, 0.39 mmol), **2c** (114 mg, 0.46 mmol) and K_2_CO_3_ (590 mg, 4.3 mmol). Yield: 86 mg (0.17 mmol, 44%); R*f* = 0.45 (ethyl acetate/*n*-hexane, 1:1); colorless oil. *υ*_max_(ATR)/cm^−1^ 2938, 2870, 2839, 1730, 1583, 1515, 1462, 1415, 1372, 1327, 1305, 1255, 1236, 1173, 1123, 1022, 1005, 939, 891, 836, 811, 769, 734, 659, 630. ^1^H NMR (300 MHz, CDCl_3_): *δ* = 1.23 (t, 3H, *J* = 7.1 Hz), 1.4–1.5 (m, 2H), 1.6–1.8 (m, 2H), 1.8–1.9 (m, 2H), 2.30 (d, 2H, *J* = 7.3 Hz), 3.77 (s, 6H), 3.86 (s, 3H), 3.87 (s, 3H), 3.91 (t, 2H, *J* = 6.6 Hz), 4.10 (q, 2H, *J* = 7.1 Hz), 6.86 (d, 1H, *J* = 8.4 Hz), 6.90 (s, 2H), 7.11 (s, 1H), 7.20 (d, 1H, *J* = 8.4 Hz), 7.89 ppm (s, 1H). ^13^C NMR (75.5 MHz, CDCl_3_): *δ* = 14.2, 24.7, 25.6, 28.8, 34.2, 56.0, 56.1, 60.2, 60.9, 68.8, 105.0, 111.5, 111.8, 120.2, 121.3, 127.7, 133.6, 138.0, 145.7, 148.5, 149.2, 150.1, 153.3, 173.6 ppm. *m/z* (%) 499 (100) [M^+^], 484 (22), 454 (15), 357 (12), 342 (17), 249 (15), 154 (53), 143 (99), 115 (27), 97 (58), 69 (43).

### 4.5. General Procedure for the Synthesis of Compounds **4a**–**i**

The corresponding compound **3** (0.20 mmol) was dissolved in CH_2_Cl_2_/MeOH (9 mL, 1:2), hydroxylamine (50% in water, 0.5 mL, 15 mmol) and NaOH (200 mg, 5 mmol) were added and the reaction mixture was stirred at room temperature for 1 h. The solvent was removed, the residue was dissolved in water and adjusted to pH 7–8 with acetic acid. The aqueous phase was extracted with ethyl acetate (2 × 50 mL), dried over Na_2_SO_4_ and concentrated in vacuum. The solid residue was recrystallized from CH_2_Cl_2_/*n*-hexane.

**N-Hydroxy-4-[1-methoxy-4-(4’-(3’’-chloro-4’’,5’’-dimethoxyphenyl)oxazol-5’-yl)-2-phenoxy]butanamide (4a).** Compound **4a** was obtained from **3a** (97 mg, 0.20 mmol), hydroxylamine (50% in water, 0.5 mL, 15 mmol) and NaOH (200 mg, 5 mmol). Yield: 64 mg (0.14 mmol, 70%); colorless solid of mp 171–172 °C. *υ*_max_(ATR)/cm^−1^ 3101, 2942, 2868, 2843, 1655, 1593, 1566, 1516, 1492, 1463, 1441, 1401, 1364, 1328, 1258, 1242, 1228, 1206, 1179, 1168, 1141, 1117, 1100, 1073, 1052, 1024, 997, 941, 887, 866, 847, 808, 755, 710, 655, 625. ^1^H NMR (300 MHz, CDCl_3_): *δ* = 2.0–2.2 (m, 2H), 2.4–2.5 (m, 2H), 3.82 (s, 3H), 3.88 (s, 3H), 3.88 (s, 3H), 3.9–4.0 (m, 5H), 6.90 (d, 1H, *J* = 8.5 Hz), 7.07 (s, 1H), 7.15 (s, 1H), 7.2–7.3 (m, 3H), 7.89 ppm (s, 1H). ^13^C NMR (75.5 MHz, CDCl_3_): *δ* = 24.4, 30.5, 56.0, 56.2, 60.9, 68.4, 110.8, 111.3, 111.5, 120.6, 121.0, 121.2, 128.3, 128.7, 132.4, 145.2, 145.8, 147.8, 149.4, 149.8, 153.9, 170.6 ppm. *m/z* (%) 464 (4) [M^+^], 462 (10) [M^+^], 447 (27), 363 (32), 361 (100), 346 (19), 318 (10), 86 (12). Elemental analysis calculated (%) for C_22_H_23_ClN_2_O_7_: C 57.09, H 5.01, N 6.05. Found: C 57.06, H 5.02, N 6.04.

**N-Hydroxy-5-[1-methoxy-4-(4’-(3’’-chloro-4’’,5’’-dimethoxyphenyl)oxazol-5’-yl)-2-phenoxy]pentanamide (4b).** Compound **4b** was obtained from **3b** (90 mg, 0.18 mmol), hydroxylamine (50% in water, 0.5 mL, 15 mmol) and NaOH (200 mg, 5 mmol). Yield: 62 mg (0.13 mmol, 72%); colorless solid of mp 202 °C. *υ*_max_(ATR)/cm^−1^ 3194, 2942, 2870, 2837, 1644, 1606, 1592, 1564, 1513, 1489, 1460, 1440, 1400, 1364, 1326, 1255, 1230, 1206, 1176, 1140, 1107, 1047, 1019, 996, 939, 895, 853, 807, 759, 711, 657, 628, 607. ^1^H NMR (300 MHz, CDCl_3_): *δ* = 1.7–1.9 (m, 4H), 2.3–2.4 (m, 2H), 3.82 (s, 3H), 3.88 (s, 3H), 3.9–4.0 (m, 5H), 6.91 (d, 1H, *J* = 8.4 Hz), 7.04 (s, 1H), 7.1-7.3 (m, 3H), 7.90 ppm (s, 1H). ^13^C NMR (75.5 MHz, CDCl_3_): *δ* = 21.5, 24.4, 26.4, 32.1, 55.9, 56.2, 60.9, 69.8, 110.2, 110.8, 111.3, 111.7, 119.9, 121.2, 128.3, 128.8, 132.4, 145.1, 146.0, 148.1, 149.2, 149.4, 153.9, 170.2 ppm. *m/z* (%) 476 (3) [M^+^], 461 (42), 363 (33), 361 (100), 346 (24), 318 (16), 240 (10), 100 (41), 44 (56). Elemental analysis calculated (%) for C_23_H_25_ClN_2_O_7_: C 57.92, H 5.28, N 5.87. Found: C 57.90, H 5.26, N 5.86.

**N-Hydroxy-6-[1-methoxy-4-(4’-(3’’-chloro-4’’,5’’-dimethoxyphenyl)oxazol-5’-yl)-2-phenoxy]hexanamide (4c).** Compound **4c** was obtained from **3c** (58 mg, 0.12 mmol), hydroxylamine (50% in water, 0.5 mL, 15 mmol) and NaOH (200 mg, 5 mmol). Yield: 44 mg (0.09 mmol, 75%); colorless solid of mp 189 °C. *υ*_max_(ATR)/cm^−1^ 3180, 3120, 2997, 2939, 2865, 2837, 1652, 1595, 1567, 1513, 1491, 1464, 1435, 1399, 1363, 1327, 1305, 1257, 1228, 1206, 1192, 1175, 1141, 1110, 1074, 1049, 1017, 996, 951, 895, 870, 851, 823, 807, 777, 758, 729, 709, 657, 630. ^1^H NMR (300 MHz, CDCl_3_): *δ* = 1.4–1.5 (m, 2H), 1.6–1.7 (m, 2H), 1.7–1.8 (m, 2H), 2.1-2.2 (m, 2H), 3.8-3.9 (m, 11H), 6.90 (d, 1H, *J* = 8.4 Hz), 7.04 (s, 1H), 7.15 (s, 1H), 7.21 (d, 1H, *J* = 8.4 Hz), 7.28 (s, 1H), 7.89 ppm (s, 1H). ^13^C NMR (75.5 MHz, CDCl_3_): *δ* = 24.9, 25.5, 28.3, 32.8, 56.0, 56.2, 60.9, 68.9, 110.8, 111.3, 111.8, 119.9, 120.9, 121.3, 128.3, 129.0, 132.2, 145.0, 146.1, 148.4, 149.4, 150.0, 153.8, 170.9 ppm. *m/z* (%) 491 (5) [M^+^], 489 (14) [M^+^], 477 (23), 475 (72), 363 (23), 361 (100), 348 (13), 346 (36), 318 (23), 182 (13), 114 (45), 69 (51). Elemental analysis calculated (%) for C_24_H_27_ClN_2_O_7_: C 58.72, H 5.54, N 5.71. Found: C 58.70, H 5.56, N 5.69.

**N-Hydroxy-4-[1-methoxy-4-(4’-(3’’-bromo-4’’,5’’-dimethoxyphenyl)oxazol-5’-yl)-2-phenoxy]butanamide (4d).** Compound **4d** was obtained from **3d** (100 mg, 0.19 mmol), hydroxylamine (50% in water, 0.5 mL, 15 mmol) and NaOH (200 mg, 5 mmol). Yield: 86 mg (0.17 mmol, 90%); colorless solid of mp 140–141 °C. *υ*_max_(ATR)/cm^−1^ 3192, 2934, 2837, 1639, 1592, 1559, 1513, 1486, 1463, 1440, 1400, 1362, 1255, 1229, 1179, 1140, 1107, 1076, 1041, 1021, 994, 954, 888, 856, 844, 806, 753, 733, 697, 657, 627. ^1^H NMR (300 MHz, CDCl_3_): *δ* = 2.0–2.2 (m, 2 H), 2.4–2.5 (m, 2H), 3.82 (s, 3H), 3.87 (s, 3H), 3.9–4.0 (m, 5H), 6.91 (d, 1H, *J* = 8.5 Hz), 7.08 (s, 1H), 7.19 (s, 1H), 7.2–7.3 (m, 1H), 7.43 (s, 1H), 7.89 ppm (s, 1H). ^13^C NMR (75.5 MHz, CDCl_3_): *δ* = 24.4, 30.6, 56.0, 56.2, 60.7, 68.5, 111.3, 111.6, 117.6, 120.5, 121.1, 124.0, 129.4, 132.3, 145.9, 146.2, 147.8, 149.4, 149.8, 153.8, 170.5 ppm. *m/z* (%) 508 (7) [M^+^], 506 (8) [M^+^], 493 (17), 491 (20), 407 (66), 405 (67), 102 (26), 86 (32), 33 (100). Elemental analysis calculated (%) for C_22_H_23_BrN_2_O_7_: C 52.08, H 4.57, N 5.52. Found: C 52.06, H 4.56, N 5.50.

**N-Hydroxy-5-[1-methoxy-4-(4’-(3’’-bromo-4’’,5’’-dimethoxyphenyl)oxazol-5’-yl)-2-phenoxy]pentanamide (4e).** Compound **4e** was obtained from **3e** (110 mg, 0.21 mmol), hydroxylamine (50% in water, 0.5 mL, 15 mmol) and NaOH (200 mg, 5 mmol). Yield: 74 mg (0.14 mmol, 67%); colorless solid of mp 152–153 °C. *υ*_max_(ATR)/cm^−1^ 3186, 2940, 1641, 1593, 1563, 1515, 1487, 1401, 1361, 1327, 1257, 1231, 1181, 1162, 1143, 1106, 1074, 1040, 1022, 993, 950, 894, 858, 845, 802, 753, 700, 655, 628. ^1^H NMR (300 MHz, CDCl_3_): *δ* = 1.8–1.9 (m, 4 H), 2.3–2.5 (m, 2H), 3.82 (s, 3H), 3.87 (s, 3H), 3.9–4.0 (m, 5H), 6.90 (d, 1H, *J* = 8.5 Hz), 7.05 (s, 1H), 7.2–7.3 (m, 2H), 7.44 (s, 1H), 7.89 ppm (s, 1H). ^13^C NMR (75.5 MHz, CDCl_3_): *δ* = 24.4, 26.4, 32.1, 55.9, 56.2, 60.8, 69.8, 110.1, 111.3, 111.6, 117.6, 119.9, 121.2, 124.0, 129.5, 132.3, 146.0, 146.2, 148.1, 149.2, 149.4, 153.7, 170.3 ppm. *m/z* (%) 521 (5) [M^+^], 519 (5) [M^+^], 505 (24), 407 (100), 405 (95), 392 (24), 390 (24), 364 (12), 362 (13), 100 (93), 72 (67), 55 (54). Elemental analysis calculated (%) for C_23_H_25_BrN_2_O_7_: C 52.99, H 4.83, N 5.37. Found: C 52.97, H 4.82, N 5.35.

**N-Hydroxy-6-[1-methoxy-4-(4’-(3’’-bromo-4’’,5’’-dimethoxyphenyl)oxazol-5’-yl)-2-phenoxy]hexanamide (4f).** Compound **4f** was obtained from **3f** (80 mg, 0.15 mmol), hydroxylamine (50% in water, 0.5 mL, 15 mmol) and NaOH (200 mg, 5 mmol). Yield: 75 mg (0.14 mmol, 93%); colorless solid of mp 115–117 °C. *υ*_max_(ATR)/cm^−1^ 3190, 2938, 2865, 1647, 1591, 1558, 1512, 1485, 1463, 1400, 1362, 1325, 1254, 1229, 1203, 1176, 1139, 1107, 1042, 998, 939, 853, 807, 775, 753, 697, 658, 628. ^1^H NMR (300 MHz, CDCl_3_): *δ* = 1.4–1.5 (m, 2H), 1.6–1.7 (m, 2H), 1.7–1.8 (m, 4H), 2.2–2.3 (m, 2H), 3.80 (s, 3H), 3.8–3.9 (m, 8H), 6.89 (d, 1H, *J* = 8.5 Hz), 7.05 (s, 1H), 7.2–7.3 (m, 2H), 7.43 (s, 1H), 7.89 ppm (s, 1H). ^13^C NMR (75.5 MHz, CDCl_3_): *δ* = 25.0, 25.5, 28.3, 32.8, 56.0, 56.1, 60.8, 68.9, 111.2, 111.6, 111.7, 117.6, 119.9, 120.9, 124.1, 129.6, 132.0, 146.0, 146.1, 148.4, 149.4, 150.0, 153.7, 171.0 ppm. *m/z* (%) 536 (1) [M^+^], 534 (1) [M^+^], 521 (91), 519 (100), 407 (53), 405 (51), 392 (17), 390 (17), 364 (7), 362 (8), 282 (7), 265 (12), 243 (7), 194 (16), 167 (28), 114 (36), 69 (17), 44 (30). Elemental analysis calculated (%) for C_24_H_27_BrN_2_O_7_: C 53.84, H 5.08, N 5.23. Found: C 53.82, H 5.06, N 5.22.

**N-Hydroxy-4-[1-methoxy-4-(4’-(3’’,4’’,5’’-trimethoxyphenyl)oxazol-5’-yl)-2-phenoxy]butanamide (4g).** Compound **4g** was obtained from **3g** (119 mg, 0.25 mmol), hydroxylamine (50% in water, 0.5 mL, 15 mmol) and NaOH (200 mg, 5 mmol). Yield: 74 mg (0.16 mmol, 64%); colorless solid of mp 121–123°C. *υ*_max_(ATR)/cm^−1^ 3194, 2935, 2837, 1658, 1583, 1515, 1454, 1415, 1373, 1256, 1238, 1173, 1123, 1020, 1002, 939, 884, 838, 813, 767, 734, 658, 628. ^1^H NMR (300 MHz, CDCl_3_) *δ* 2.0–2.2 (m, 2H), 2.3–2.4 (m, 2H), 3.76 (s, 6H), 3.85 (s, 3H), 3.9–4.0 (m, 5H), 4.15 (q, 2H, *J* = 7.1 Hz), 6.8–6.9 (m, 3H), 7.05 (s, 1H), 7.2–7.3 (m, 1H), 7.90 ppm (s, 1H). ^13^C NMR (75.5 MHz, CDCl_3_): *δ* = 24.5, 30.2, 56.0, 56.2, 61.0, 68.2, 105.2, 110.0, 111.4, 120.4, 121.3, 127.8, 133.7, 145.5, 147.7, 149.3, 149.7, 153.3, 170.5 ppm. *m/z* (%) 458 (4) [M^+^], 443 (100), 428 (26), 357 (98), 342 (62), 314 (17), 236 (16), 195 (18), 151 (12), 86 (22), 44 (29). Elemental analysis calculated (%) for C_23_H_26_N_2_O_8_: C 60.26, H 5.72, N 6.11. Found: C 60.23, H 5.70, N 6.08.

**N-Hydroxy-5-[1-methoxy-4-(4’-(3’’,4’’,5’’-trimethoxyphenyl)oxazol-5’-yl)-2-phenoxy]pentanamide (4h).** Compound **4h** was obtained from **3h** (98 mg, 0.20 mmol), hydroxylamine (50% in water, 0.5 mL, 15 mmol) and NaOH (200 mg, 5 mmol). Yield: 72 mg (0.15 mmol, 76%); colorless solid of mp 118–120°C. *υ*_max_(ATR)/cm^−1^ 3191, 2937, 2870, 2843, 1657, 1584, 1515, 1454, 1415, 1373, 1306, 1255, 1239, 1173, 1123, 1022, 838, 811, 768, 734, 658, 629. ^1^H NMR (300 MHz, CDCl_3_): *δ* = 1.8–1.9 (m, 4H), 2.2–2.3 (m, 2H), 3.78 (s, 6H), 3.8–3.9 (m, 5H), 3.94 (s, 3H), 6.8-6.9 (m, 3H), 7.02 (s, 1H), 7.2–7.3 (m, 1H), 7.90 ppm (s, 1H). ^13^C NMR (75.5 MHz, CDCl_3_): *δ* = 24.0, 26.6, 32.0, 55.9, 56.2, 61.0, 69.5, 105.3, 110.4, 111.2, 119.9, 121.5, 127.9, 133.7, 137.8, 145.6, 148.0, 149.1, 149.3, 153.3, 170.1 ppm. *m/z* (%) 472 (3) [M^+^], 457 (100), 442 (10), 357 (73), 342 (34), 314 (11), 100 (17), 44 (12). Elemental analysis calculated (%) for C_24_H_28_N_2_O_8_: C 61.01, H 5.97, N 5.93. Found: C 61.04, H 5.95, N 5.90.

**N-Hydroxy-6-[1-methoxy-4-(4’-(3’’,4’’,5’’-trimethoxyphenyl)oxazol-5’-yl)-2-phenoxy]hexanamide (4i).** Compound **4i** was obtained from **3i** (73 mg, 0.15 mmol), hydroxylamine (50% in water, 0.5 mL, 15 mmol) and NaOH (200 mg, 5 mmol). Yield: 52 mg (0.11 mmol, 73%); colorless solid of mp 114–116 °C. *υ*_max_(ATR)/cm^−1^ 3211, 2936, 2870, 2837, 1651, 1584, 1515, 1455, 1414, 1372, 1331, 1306, 1255, 1238, 1173, 1122, 1019, 1001, 939, 892, 836, 810, 767, 732, 658, 628. ^1^H NMR (300 MHz, CDCl_3_): *δ* = 1.3–1.4 (m, 2H), 1.5–1.7 (m, 4H), 2.1–2.2 (m, 2H), 3.76 (s, 6H), 3.8–3.9 (m, 8H), 6.8-6.9 (m, 3H), 7.03 (s, 1H), 7.2–7.3 (m, 1H), 7.90 ppm (s, 1H). ^13^C NMR (75.5 MHz, CDCl_3_): *δ* = 25.0, 25.4, 28.3, 32.8, 56.0, 56.1, 61.0, 68.8, 105.2, 111.5, 111.6, 119.8, 121.2, 127.9, 133.4, 137.6, 145.7, 148.2, 149.3, 149.8, 153.2, 171.0 ppm. *m/z* (%) 486 (18) [M^+^], 471 (100), 456 (17), 443 (11), 357 (48), 342 (38), 193 (17), 114 (13), 44 (21). Elemental analysis calculated (%) for C_25_H_30_N_2_O_8_: C 61.72, H 6.22, N 5.76. Found: C 61.70, H 6.21, N 5.74.

### 4.6. Ester Hydrolysis of **3g** to Carboxylic Acid **4j**

**4-[1-Methoxy-4-(4’-(3’’,4’’,5’’-trimethoxyphenyl)oxazol-5’-yl)-2-phenoxy]butyric acid (4j).** Compound **3g** (119 mg, 0.25 mmol) was dissolved in MeOH (10 mL), aqueous NaOH (1 M, 10 mL) was added and the reaction mixture was stirred at room temperature for 24 h. The solution was acidified with aqueous HCl (1 M, to pH < 2) and the resulting precipitate was collected, washed with water, and dried in vacuum. Yield: 75 mg (0.17 mmol, 68%); colorless solid of mp 209–210 °C. *υ*_max_(ATR)/cm^−1^ 3134, 2940, 2873, 2835, 2538, 1728, 1606, 1586, 1519, 1474, 1446, 1414, 1372, 1316, 1274, 1251, 1237, 1196, 1173, 1147, 1128, 1112, 1068, 1043, 1031, 1022, 1007,962, 946, 883, 855, 840, 804, 769, 761, 736, 674, 654, 630, 623. ^1^H NMR (300 MHz, CDCl_3_): *δ* = 2.0–2.2 (m, 2H), 2.56 (t, 2H, *J* = 7.2 Hz), 3.76 (s, 6H), 3.86 (s, 3H), 3.87 (s, 3H), 3.98 (t, 1H, *J* = 6.3 Hz), 6.86 (d, 1H, *J* = 8.5 Hz), 6.88 (s, 2H), 7.12 (s, 1H), 7.22 (d, 1H, *J* = 8.5 Hz), 7.91 ppm (s, 1H). ^13^C NMR (75.5 MHz, CDCl_3_): *δ* = 24.2, 30.2, 56.0, 56.1, 60.9, 67.8, 105.1, 111.6, 112.1, 120.5, 121.3, 127.6, 133.5, 137.9, 145.6, 148.2, 149.3, 150.1, 153.3, 177.0 ppm. *m/z* (ESI, %) 466.2 (27) [M^+^ + Na], 444.2 (100) [M^+^]. Elemental analysis calculated (%) for C_23_H_25_NO_8_: C 62.30, H 5.68, N 3.16. Found: C 62.26, H 5.65, N 3.08.

### 4.7. Biological Evaluations

#### 4.7.1. Cell Lines and Culture Conditions

HT-29 (ACC-299), HCT-116 (ACC-581) and DLD-1 (ACC-278) colon carcinoma, MCF-7 (ACC-115) breast carcinoma, KB-V1 (ACC-149), 518A2 (Department of Radiotherapy and Radiobiology, University Hospital Vienna) melanoma and Ea.Hy926 (ATCC no. CRL-2922) HUVEC derived endothelial hybrid cells line were grown in Dulbecco’s Modified Eagle Medium (DMEM; Biochrom), high glucose supplemented with 10% (*v*/*v*) fetal bovine serum (FBS; Biochrom.) and 1% (*v*/*v*) Antibiotic-Antimycotic solution (anti-anti; Thermo Scientific). HDFa human dermal fibroblasts (ATCC: PCS-201-012™) were grown in DMEM supplemented with 10% FBS, 1% anti-anti and 2 mM glutamine. The cells were incubated at 37 °C, 5% CO_2_, 95% humidified atmosphere. By repeated addition of topotecan or vinblastine at the maximum tolerated dose to the cell medium of MCF-7 and KB-V1 cells, the cells were rendered multidrug-resistant, indicated as MCF-7^Topo^ and KB-V1^Vbl^, respectively. They were serially passaged following trypsinization by using 0.05% trypsin/0.02% EDTA (Biochrom). Mycoplasma contamination was routinely monitored, and only mycoplasma–free cultures were used.

#### 4.7.2. MTT Assay

MTT [3-(4,5-dimethylthiazol-2-yl)-2,5-diphenyltetrazolium bromide] (ABCR) was used to determine the cytotoxicity of test compounds as previously described [53]. Briefly, HDFa human dermal fibroblasts and Ea.Hy926 endothelial hybrid cells (both 1 × 10^5^ cells/mL, 100 µL/well), 518A2 melanoma, KB-V1^Vbl^ cervix carcinoma, MCF-7^Topo^ breast carcinoma, HT-29, DLD-1 and HCT-116 colon carcinoma (all 5 × 10^4^ cells/mL, 100 µL/well) were grown in 96-well culture plates for 24 h. Then, various concentrations of the test compounds were added and the cells were incubated for 24-72 h at 37 °C. After adding 12.5 µL of a 0.5% MTT solution in PBS (final concentration 0.05%) to cell medium, microplates were incubated for 2 h and subsequently swiftly turned to discard the medium. The precipitate of formazan crystals was then dissolved in a 10% solution of SDS in DMSO containing 0.6% acetic acid. To ensure complete dissolution of formazan, microplates were incubated for at least 4 h at 37 °C. Finally, the absorbance at λ = 570 nm (formazan) and 630 nm (background) was measured using a microplate reader (Tecan F200). All experiments were carried out in quadruplicate and the percentage of viable cells was calculated as the mean ± SD with controls set to 100%. Selected graphs of MTT assays can be found in the Supporting Information (Figure S8).

#### 4.7.3. Tubulin Polymerization Assay

An amount of 50 µL of Brinkley’s buffer 80 (BRB80) supplemented with 20% glycerol and 3 mM GTP was given in a black 96-well half-area plate with clear bottom. Then, test compounds **4d**–**f** (final concentration: 5 µM, or 10 µM) or vehicle (DMSO) were added. After adding 50 µL tubulin in BRB80 (10 mg/mL) was pipetted in the wells and immediately placed in a pre-heated microplate reader (Tecan). The polymerization was determined turbidimetrically at 37 °C by measuring the absorption at 340 nm for 120 min in intervals of 5 min. Values were normally distributed for each group (Shapiro–Wilk test, *p* > 0.05) and there was homogeneity of variance (Levene’s test, *p* > 0.05). The one-tailed Dunnett post-hoc test revealed significant inhibition of tubulin polymerization (*p* ≤ 0.001) for CA-4 and **4d** compared to controls.

#### 4.7.4. Immunofluorescence Staining of Microtubule Cytoskeleton

518A2 melanoma cells (5 × 10^4^ cells/mL, 500 µL/well) or Ea.Hy926 endothelial hybrid cells (7.5 × 10^4^ cells/mL, 500 µL/well) were seeded in 24-well plates on small glass coverslips and grown for 24 h. Then, the cells were exposed to the test compounds for 24 h. After washing the cells once with PBS, they were fixed in 3.7% formaldehyde in PBS (20 min, rt), and permeabilized and blocked in 1% BSA and 0.1% triton X-100 in PBS (30 min, rt). Then, the cells were incubated with monoclonal mouse anti-alpha-tubulin antibody (1 h, 37 °C). After washing the cells for three times with PBS, the cells were exposed to the secondary anti-mouse 488 antibody conjugate (1 h, rt, in the dark). Then, the cells were washed for three times with PBS and once with water. The glass coverslips were mounted in 4-88 based mounting medium supplemented with 1 µg/mL DAPI for counterstaining the nuclei and 2.5% DABCO. Alterations of the microtubule were documented by a Zeiss Imager A1 AX10 fluorescence microscope (400× magnification).

#### 4.7.5. HDAC Inhibition

The HDAC inhibitory potential of the novel compounds was determined by utilizing the deacetylase activity of recombinant human HDAC1 (HDAC1 Inhibitor Screening Assay Kit; Cayman Chemicals) or HDAC6 (HDAC6 Inhibitor Screening Kit (Fluorometric); Biovision) towards the corresponding synthetic acetylated-peptide substrates resulting in the release of a fluorescent product. The assays were performed according to manufacturer’s description of the in commercially available assay kits. The fluorescence intensity (HDAC1: λ_ex_ = 352 nm, λ_em_ = 452 nm; HDAC6: λ_ex_ = 380 nm, λ_em_ = 510 nm) as a measure of enzyme activity was measured at 37 °C with a microplate reader (Tecan). The IC_50_ values were derived from dose-response curves and are expressed as the means ± SD of two independent experiments. A two-tailed t-test was performed, revealing significant differences (*p* < 0.0001) in HDAC6-inhibition for **4e**–**f** compared with **4d** as well as for **4d** and **4f** compared with **4e** and in HDAC1-inhibition for **4d**–**e** compared with **4f**. The difference in HDAC1 IC_50_ values of **4d** compared with **4e** were not significant.

#### 4.7.6. Western Blot Analyses

For the microtubule acetylation blots, 518A2 melanoma cells (5 × 10^4^ cells/mL, 3 mL/well) were grown in 6-well plates for 24 h and then incubated with vehicle (DMSO), SAHA (10 µM), or **4f** (4, 5, and 6 µM) for 24 h. After harvesting the cells by trypsination, they were pelleted by centrifugation (300× g, 5 min) followed by cell lysis in 100 µL lysis buffer (20 mM DTT, 200 µM sodium vanadate, 50 mM Tris/HCl, 1% triton X-100, 150 mM NaCl, pH 7.4) for 10 min on ice. The cell lysates were mixed with 100 µL of 2× Laemmli buffer (125 mM Tris-HCl, 4% SDS, 20% glycerol, 10% β-mercapto-ethanol, pH 6.8) and boiled (95 °C, 10 min). Equal amounts of total protein were subjected to 10% SDS-polyacrylamide gel electrophoresis (SDS-PAGE) and transferred to polyvinylidene difluoride membranes (PVDF, Carl Roth). For subsequent analysis of acetylated protein and of alpha-tubulin which was used as a loading control, membranes were blocked and incubated with primary antibody solutions in 5% bovine serum albumin in 1× TBS or 5% milk powder in PBS, respectively. The protein bands were visualized by chemoluminescence (secondary antibody-HRP conjugates, ECL detection system; Cell Signaling) using a LAS-3000 imager (Fujifilm).

For the cell cycle protein blots, 518A2 melanoma cells (2 × 106 cells/mL, 10 mL/well) were grown in 10 cm dishes for 24 h and then incubated with **4d** (0.15–4 µM) or SAHA (5 µM) for 24 h. After harvesting and lysing of the cells, western blotting was performed as described before [54]. For subsequent analysis, the protein-loaded membranes were incubated with antibodies directed against p21 (Abcam ab109199), p27 (Abcam ab109199), cyclin D1 (Cell Signaling 29225) and GADPH (Santa Cruz sc25778) in 1:1000 dilutions. After incubation with horseradish peroxidase-coupled anti-IgG antibodies (1:10,000; Amersham), the blot was developed using the Celvin-S developer (Biostep) and the software SnapAndGo 1.8.1. 

#### 4.7.7. Immunofluorescence Staining of F-Actin

518A2 melanoma cells (5 × 10^4^ cells/mL, 500 µL/well) were seeded in 24-well plates on small glass coverslips and grown for 24 h at 37 °C. Then, the cells were treated with **4d** (0.5 µM), **4e** (1.5 µM), **4f** (4 µM), and vehicle (DMSO) for 24 h. After washing the cells once with PBS at 37 °C, they were fixed with 3.7% formaldehyde solution in PBS (pH 7.0) for 10 min at room temperature. Then, the cells were washed with PBS and permeabilized with 0.5% Triton X-100 in PBS for 5 min at room temperature. After washing with PBS, the cells were incubated with 200 µL of a 0.1 µM Acti-stain^TM^ 488 phalloidin solution in PBS for 30 min at room temperature in the dark. Then, the cells were washed three times with PBS and once with water, and the glass coverslips mounted in 4-88-based mounting medium containing 1 µg/mL DAPI for counterstaining the nuclei and 2.5% DABCO. The effects on the actin cytoskeleton were documented by a Zeiss Imager A1 AX10 fluorescence microscope (400× magnification).

#### 4.7.8. Cell Cycle Analysis

The 518A2 melanoma cells (3 mL/well; 5 × 10^4^ cells/mL) were grown on 6-well tissue culture plates for 24 h. After treatment with **4d** (150, and 200 nM), **4e** (0.8, and 1 µM), **4f** (2, and 3 µM), or DMSO (control) for another 24 h, cells were fixed (70% EtOH, 24 h, 4 °C), washed with PBS and incubated with propidium iodide (PI; Carl Roth) staining solution (50 µg/mL PI, 0.1% sodium citrate, 50 µg/mL RNase A in PBS) for 30 min at 37 °C. The fluorescence intensity of 10,000 single cells was measured at λ_em_ = 570 nm (λ_ex_ = 488 nm laser source) with a Beckmann Coulter Cytomics FC 500 flow cytometer. The percentages of cells in the different phases of the cell cycle (G1, S and G2/M phase) were determined using the CXP Analysis software (Beckmann Coulter). The percentage of apoptotic cells was derived from sub-G1 peaks. Data was normally distributed for each group (Shapiro–Wilk test, *p* > 0.05). Since homogeneity of variance (Levene’s test, *p* < 0.05) was violated, a correction was calculated for Anova (Welch test). The two-tailed Games–Howell’s post-hoc test revealed the significant alteration (*p* ≤ 0.05) of the population of cells in G1 in 518A2 melanoma cells treated with **4d**–**f** compared with vehicle-treated controls.

#### 4.7.9. In Vivo Toxicity

In vivo toxicity of **4d** was studied in nude mice (Charles River Laboratories, Sulzfeld, Germany). These experiments were carried out following the institutional guidelines. **4d** was formulated in 10% Tween80/10% ethanol/80% saline for administration. One mouse (32 g) was treated once with 1 × 100 mg/kg body weight (i.p.), another mouse (30 g) was treated once with 1 × 200 mg/kg body weight (orally) of **4d** and then both mice were observed for two weeks. The body weight of the mice was assessed daily under therapy.

### 4.8. In Silico Evaluation

#### 4.8.1. Proteins and Compound Structures

The protein structures for tubulin, HDAC1, and HDAC6 used in the docking studies were obtained from the Protein Data Bank (Table 7, PDB, www.rcsb.org, accessed 6 March 2018) [55]. One representative per protein was selected for the docking studies based on the following criteria: The protein should be co-crystallized with a ligand most similar to the ligands from which the studied hybrid compounds **4d**–**f** were derived (combretastatin A-4 for tubulin and hydroxamic acids for HDACs) [56]. Among these, the structures with the best resolutions were selected.

#### 4.8.2. Initial Docking with LeadIT/FlexX

To generate initial predictions of binding positions, called docking poses, FlexX (included in LeadIT 2.2.0, which was kindly provided by BioSolveIT, www.biosolveit.de/LeadIT), was used [57]. The PDB structures were prepared as follows: For tubulin, chains A and B of 5LYJ, for HDAC1, chain B of 5ICN, and for HDAC6, chain A of 5EDU were chosen [47,50,51]. The binding pockets were defined with the co-crystallized ligands as a reference ligand, including all amino acids within a radius of 10 Å for the HDAC structures and 6.5 Å for tubulin. The metal coordination of the zinc ion in both HDAC structures was set to ’spherical’ and zinc was defined as an essential pharmacophore for guided docking. All other settings for the receptor definition were used as default. The docking library contained compounds **4d**–**f** as well as the crystal structure’s original ligand. For HDAC1 and HDAC6 vorinostat, a known HDAC inhibitor, was added to the library. The docking strategy, scoring and chemical parameters were kept as default. Only the maximum number of solutions per iteration and the maximum number of solutions per fragmentation were increased to 1000 steps each.

#### 4.8.3. Pose Optimization with SeeSAR

Final pose optimization and affinity estimation was performed with SeeSAR 6.1, also kindly provided by BioSolveIT (www.biosolveit.de/SeeSAR). The best poses from the LeadIT docking were imported and were used for binding site definition. For each compound, 10 new poses were generated, and all were evaluated with the built in HYDE scoring function [58]. The poses with the best estimated affinities were chosen for further analysis.

#### 4.8.4. Additional Software Used

PyMOL was used to analyze, compare, and visualize the binding pose predictions, as well as to create the 3D images [59].

## Figures and Tables

**Table 1:** Inhibitory concentrations IC_50_^1^ (µM) of **3g**, **4a–j**, SAHA, and CA-4 in cancer cells.

	518A2 (24 h)	518A2 (72 h)	HT-29 (24 h)	HT-29 (72 h)	DLD-1 (72 h)	HCT-116 (72 h)	KB-V1^Vbl^ (72 h)	MCF-7^Topo^ (72 h)	Ea.Hy926 (72 h)	HDFa (72 h)
**3g**	>100	>50	>100	>50	-^c^	-	-	-	-	-
**4a**	1.3 ± 0.4	0.11 ± 0.01	2.5 ± 0.4	2.9 ± 0.3	0.34 ± 0.01	0.21 ± 0.01	0.15 ± 0.02	0.66 ± 0.14	0.0012 ± 0.0006	-
**4b**	10.4 ± 5.1	1.0 ± 0.1	4.2 ± 0.2	3.6 ± 0.1	3.2 ± 0.1	2.4 ± 0.1	4.0 ± 0.2	2.9 ± 0.8	0.57 ± 0.07	-
**4c**	9.4 ± 1.4	2.2 ± 0.3	4.6 ± 0.3	4.9 ± 0.3	3.3 ± 0.5	6.5 ± 0.3	18.4 ± 3.4	6.1 ± 0.7	7.5 ± 0.5	-
**4d**	0.71 ± 0.05	0.11 ± 0.01	1.3 ± 0.3	1.4 ± 0.2	0.29 ± 0.02	0.13 ± 0.01	0.48 ± 0.06	2.8 ± 0.3	0.018 ± 0.004	23.9 ± 1.6
**4e**	2.4 ± 0.3	0.80 ± 0.07	2.1 ± 0.6	1.9 ± 0.2	2.1 ± 0.4	2.2 ± 0.3	2.0 ± 0.3	3.4 ± 0.2	0.66 ± 0.01	>100
**4f**	7.6 ± 0.2	6.4 ± 0.4	6.6 ± 0.6	14.2 ± 1.6	4.1 ± 0.3	6.3 ± 0.4	7.1 ± 0.2	8.3 ± 0.7	0.41 ± 0.04	>50
**4g**	4.4 ± 0.1	0.64 ± 0.07	5.0 ± 0.2	2.8 ± 0.2	0.92 ± 0.04	0.92 ± 0.12	2.7 ± 0.2	9.3 ± 0.8	0.41 ± 0.04	-
**4h**	21.6 ± 3.9	5.6 ± 0.6	53.9 ± 12.0	6.6 ± 0.6	5.8 ± 0.4	3.4 ± 0.4	11.1 ± 1.6	39.6 ± 5.9	3.4 ± 0.2	-
**4i**	6.9 ± 2.5	3.3 ± 0.3	47.6 ± 10.6	1.7 ± 0.1	1.3 ± 0.1	0.61 ± 0.07	9.0 ± 0.3	15.8 ± 1.4	1.7 ± 0.1	-
**4j**	>100	>50	>100	>100	-	-	-	-	-	-
**SAHA^2^**	18.7 ± 0.1	1.8 ± 0.1	1.9 ± 0.3	1.8 ± 0.1	-	0.9 ± 0.1	13.1 ± 0.8	13.5 ± 0.7	1.9 ± 0.2	-
**CA-4^2^**	-	0.018 ± 0.007	-	3.6 ± 0.1	-	0.0026 ± 0.0002	-	0.50 ± 0.20	0.011 ± 0.002	-

^1^ Values are derived from dose-response curves obtained by determining the percentage of viable cells (human 518A2 melanoma, HT-29, HCT-116 and DLD-1 colon carcinomas, KB-V1^Vbl^ cervix carcinoma, and MCF-7^Topo^ breast adenocarcinoma as well as to Ea.Hy926 endothelial hybrid cells and non-malignant HDFa dermal fibroblasts) relative to vehicle treated controls after 24 h or 72 h treatment with the test compounds using MTT-assays; values are the means ± SD of four independent experiments. ^2^ Values taken from earlier publications, ref 27, 33, and 34. *^c^*- = not determined.

**Table 2:** HDAC inhibition [IC_50_ (µM)]^1^ by compounds 4**d**–**f**.

	4d	4e	4f	SAHA	Tubacin
**HDAC1**	4.0 ± 0.1	3.8 ± 0.1	0.49 ± 0.05	0.44 ± 0.04	-
**HDAC6**	13.8 ± 0.2	3.5 ± 0.1	0.32 ± 0.02	0.15 ± 0.004	0.38 ± 0.03

^1^ IC_50_ values are derived from dose-response curves obtained by measuring the percentage of deacetylated, fluorogenic substrate relative to DMSO controls. Results show inhibition of recombinant HDAC1 and HDAC6 by compounds **4d–f**, pan-HDAC inhibitor SAHA and HDAC6-specific inhibitor tubacin as determined by conversion of a HDAC substrate to a fluorophore. Values are the means ± SD of two independent experiments.

**Table 3:** Effect of **4d–4f** on the cell cycle of 518A2 melanoma cells ^1^.

	Sub-G1	G1	S	G2/M
**Control**	4.9 ± 2.0	49.7 ± 1.0	19.3 ± 0.9	26.1 ± 2.3
**4d** (0.15 µM)	8.9 ± 1.5	43.0 ± 0.9	19.2 ± 1.4	28.9 ± 2.7
**4d** (0.2 µM)	14.4 ± 2.8	33.9 ± 3.4	16.3 ± 1.1	35.4 ± 2.4
**4e** (0.8 µM)	5.1 ± 1.2	43.1 ± 1.3	19.2 ± 0.7	32.6 ± 1.7
**4e** (1 µM)	10.8 ± 4.4	35.4 ± 1.1	19.0 ± 0.3	34.9 ± 3.5
**4f** (2 µM)	5.6 ± 0.8	53.9 ± 0.8	18.3 ± 0.7	22.2 ± 0.5
**4f** (3 µM)	6.3 ± 0.9	54.2 ± 0.5	16.9 ± 0.6	22.5 ± 0.3

^1^ Effect of **4d** (0.15 and 0.2 µM), **4e** (0.8 and 1 µM), **4f** (2 and 3 µM), or vehicle (DMSO) on the proportions of 518A2 melanoma cells in G1, S, and G2/M phase of the cell cycle as well as the percentages of apoptotic cells in sub-G1 as determined by flow cytometry after PI staining. Cells were treated with the test compound for 24 h. Values are the means ± SD of at least three independent experiments.

**Table 4:** SeeSAR Docking results including estimated affinities (EA) and hydrogen bonds for Tubulin structure 5LYJ ordered by best estimated affinities.

	EA (SeeSAR)	Hydrogen Bonds (SeeSAR)
**CA-4**	0.11–11 µM	
**4d**	2.12–221 µM	Asn349
**4e**	10–1019 µM	Asn349
**4f**	24–2350 µM	Asn349, Ser178 (Chain A)

EA ranges mark the lower and upper bound of the estimated affinities (±1.5 log-units). Note that the estimated affinity values can be quantitatively compared within one protein structure but not necessarily across different proteins.

**Table 5:** SeeSAR docking results including estimated affinities (EA) and hydrogen bonds for HDAC6 structure 5EDU ordered by best estimated affinities.

	EA (SeeSAR)	Hydrogen Bonds (SeeSAR)
**Trichostatin A**	< 0.02 µM	**His610, Gly619, His611, Tyr782**
**4f**	0.32–32 µM	**His610, Gly619**
**4e**	0.40–39 µM	**His610, Gly619**
**Vorinostat**	1.26–125 µM	**His610, Gly619,** His651
**4d**	28–2808 µM	**His610, Gly619,** His651

EA ranges mark the lower and upper bound of the estimated affinities (±1.5 log-units). Amino acids forming hydrogen bonds with hydroxamic acid are shown in bold. Note that the estimated affinity values can be quantitatively compared within one protein structure but not necessarily across different proteins.

**Table 6:** SeeSAR docking results including estimated affinities (EA) and hydrogen bonds for HDAC1 structure 5ICN ordered by best estimated affinities.

	EA (SeeSAR)	Hydrogen Bonds (SeeSAR)
**H4K16Hx**	< 0.81 µM	**Tyr303, His140, His141,** Asn95, Glu98, Asp99, Cys100, Gly149
**4f**	56–5.543 µM	**Tyr303, His141,** Asn95, Gly149
**Vorinostat**	1.049–104.186 µM	**His140,** Gly149
**4e**	3.814–378.935 µM	**Gly301,** Asn95
**4d**	Not scored	–

EA ranges mark the lower and upper bound of the estimated affinities (±1.5 log-units). Amino acids forming hydrogen bonds with hydroxamic acid are shown in bold. Note that the estimated affinity values can be quantitatively compared within one protein structure but not necessarily across different proteins.

**Table 7:** Selection of PBD structures used for docking.

	PDB	Chain	Res.	Original Ligand
**Tubulin**	5LYJ	A&B	2.4 Å	Combretastatin A-4
**HDAC1**	5ICN	B	3.3 Å	H4K16Hx
**HDAC6**	5EDU	A	2.8 Å	Trichostatin A

## Supplementary Materials

Supplementary Materials can be found at http://www.mdpi.com/1422-0067/20/2/383/s1.

## Abbreviations

CA-4	Combretastatin A-4
DMEM	Dulbecco’s modified Eagle medium
EMT	Epithelial-to-mesenchymal transition
FBS	Fetal bovine serum
HDAC	Histone deacetylase
HDACi	HDAC inhibitor
HRP	Horseradish peroxidase
MDA	Microtubule disrupting agent
PI	Propidium iodide
ROS	Reactive oxygen species
SAHA	Suberoyl anilide hydroxamic acid
SAR	Structure activity relationship
SD	Standard deviation
VDA	Vascular-disrupting agents
ZBG	Zinc binding group
