# Supplementary material for: Oxazole-Bridged Combretastatin A-4 Derivatives with Tethered Hydroxamic Acids: Structure–Activity Relations of New Inhibitors of HDAC and/or Tubulin Function
Source: Int J Mol Sci. 2019 Jan 17;20(2):383. doi: 10.3390/ijms20020383 (PMC6359144; doi:10.3390/ijms20020383)
Supplement: Supplementary file 1 [file ijms-20-00383-s001.pdf]

-Supporting Information-

Oxazole-bridged combretastatin A-4 derivatives with tethered hydroxamic acids: Structure-activity relations of new inhibitors of HDAC and/or tubulin function

Florian Schmitt [1], Lisa Chiara Gosch [2,3], Alexandra Dittmer [2], Matthias Rothemund [1], Thomas Mueller [4], Rainer Schobert [1], Bernhard Biersack [1,*], Andrea Volkamer [3] and Michael Höpfner [2,*]

[1] Department of Chemistry, University of Bayreuth, Universitaetsstrasse 30, 95447 Bayreuth, Germany; florian1.schmitt@uni-bayreuth.de (F.S.), matthias.rothemund@uni-bayreuth.de (M.R.), rainer.schobert@uni-bayreuth.de (R.S.)

[2] Institute of Physiology, Charité–Universitätsmedizin Berlin, Charitéplatz 1, 10117 Berlin, Germany; lisa-chiara.gosch@charite.de (L.C.G.), alexandra.dittmer@charite.de (A.D.)

[3] In Silico Toxicology Group, Institute of Physiology, Charité–Universitätsmedizin Berlin, Charitéplatz 1, 10117 Berlin, Germany; andrea.volkamer@charite.de

[4] Internal Medicine IV, University Hospital Halle (Saale), Ernst-Grube-Str. 40, 06120 Halle, Germany; thomas.mueller@medizin.uni-halle.de

* Correspondence: bernhard.biersack@yahoo.com (B.B.); michael.hoepfner@charite.de (M.H.); Tel.: +49-921-55-2673 (B.B.); Tel: +49-030-450-528-515(M.H.);

Table of content

Material and Methods. S1

DCFH-DA assay. S1

Immuofluorescence staining of acetylated alpha-tubulin. S2

Wound healing assay. S2

Results S3

Effect on the microtubule cytoskeleton S3

Effect on the intracellular ROS levels. S3

Effect on the acetylation of alpha-tubulin. S3

Effect on migratory activity. S5

Effect on mouse weight S6

Graphs of MTT assays S7

S1

Material and Methods

DCFH-DA assay.

The assay was performed as previously described. Briefly, 518A2 melanoma cells (1 × 105 cells/mL, 100 µL/well) were grown in a black 96-well plate for 24 h. Then, the cell medium was exchanged for serum-free DMEM supplemented with 20 µM DCFH-DA and incubated for 30 min at 37 °C. After washing the cells twice with 100 µL PBS, the cells were treated with the test compounds 4d-f (10 µM), and vehicle (DMSO) in serum-free DMEM for 1 h at 37 °C. Then, the cells were washed twice with 100 µL PBS and subsequently placed in a microplate reader (TECAN). The DCF-fluorescence (λex = 485 nm, λem = 535 nm) as a measure of ROS production was determined and that of vehicle treated controls set to 100%. Values exceeding 100% indicate a drug-induced ROS generation. The values of six independent assays were depicted as the means ± SD. There were no outliers and the data fulfilled variance homogeneity (Levene's test, $p > 0.05$). The one-tailed Dunnett post-hoc test revealed the significant elevation ($p < 0.05$) of ROS in cells treated with 4d-f compared to vehicle-treated control.

Immuofluorescence staining of acetylated alpha-tubulin.

518A2 melanoma cells (5 × 104 cells/mL, 500 µL/well) were grown on glass coverslips in 24-well plates for 24 h. Then, the cells were treated with 4f (4 µM), SAHA (10 µM), or vehicle (DMSO) for 24 h. After washing the cells with PBS, they were fixed in 3.7% formaldehyde in PBS (20 min, rt) followed by blocking and permeabilization in TBS supplemented with 5% BSA and 0.1% triton X-100 (30 min, rt). Then the cells were incubated with monoclonal rabbit anti-acetyl-alpha-tubulin antibody (2 h, 37 °C). After washing the cells for three times with PBS, they were incubated with a secondary anti-rabbit 488 antibody conjugate in the dark (2 h, rt). Then, the cells were washing again for three times with PBS and once with water. The glass coverslips were mounted in 4-88 based mounting medium containing 1 µg/mL DAPI and 2.5% DABCO. Pictures were taken by using a Zeiss Imager A1 AX10 fluorescence microscope (400× magnification).

Wound healing assay.

518A2 melanoma cells (1 × 10^5 cells/mL, 500 µL/well) were grown in 24-well plates for 24 h so that the cells have arranged in a sub-confluent monolayer. Then, a strip of cells was scraped off by using a 100 µL pipette tip. The medium containing the detached cells was aspirated and exchanged for new medium. Then, the cells were treated with vehicle (DMSO), **4d** (0.2 µM), **4e** (1.2 µM), and **4f** (3.8 µM) and wound re-closure was documented subsequently and 24 h after application by using a light microscope (Zeiss Axiovert 135, 100× magnification). The open image area after 24 h (%) compared to that at 0 h was quantified from three independent experiments by using the imageJ 1.50i software. Values were normally distributed for each group (Shapiro-Wilk test, $p > 0.05$) and there was homogeneity of variance (Levene's test, $p > 0.05$). The one-tailed Dunnett post-hoc test revealed the significant reduction ($p \leq 0.001$) of wound healing of cells treated with **4d-f** compared to vehicle.

Results

Effect on the microtubule cytoskeleton.

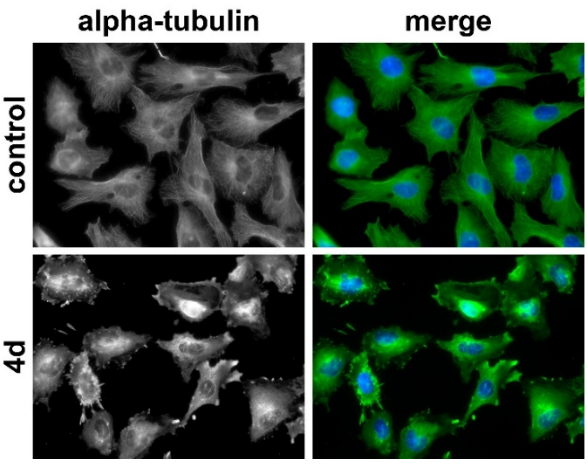

Figure S1. Effect of 4d (0.2 µM), or vehicle (DMSO) on the organization of microtubule cytoskeleton in Ea.Hy926 endothelial hybrid cells after 24 h incubation. Nuclei were counterstained with DAPI (merge, blue); alpha-tubulin (green). Pictures are representative of two independent experiments (400× magnification).

Effect on the intracellular ROS levels.

Elevated levels of reactive oxygen species (ROS) are known to trigger apoptosis and reverse chemo-resistance in tumors. Several approved chemotherapeutics exert their effect by direct or indirect promotion of ROS production which results in the induction of apoptosis. HDACi like SAHA are known to induce apoptosis by increased intracellular levels of ROS. Hence, the effect of 4d-f on the intracellular

ROS levels was tested in 518A2 melanoma cells using the DCFH-DA assay. The intracellular ROS levels were distinctly increased to levels exceeding 190% when compared to untreated controls which were set to 100% (4d: 241% ± 17; 4e: 230 ± 31; 4f: 198 ± 24).

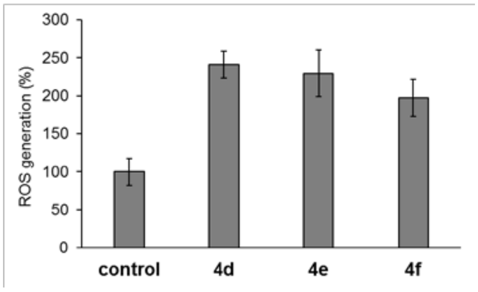

Figure S2. Effect of the test compounds 4d-f (10 μM) on the intracellular ROS levels in 518A2 melanoma cells after 1 h incubation as determined by DCFH-DA assay. Values are the means ± SD of six independent experiments with vehicle treated control cells set to 100% ROS production.

Effect on the acetylation of alpha-tubulin.

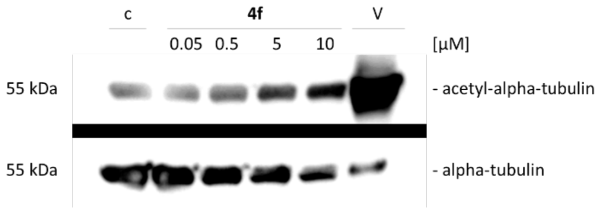

Figure S3. Compound-induced effect on the acetylation of microtubule (acetyl-alpha-tubulin) in 518A2 melanoma cells upon treatment with **4f** (0.05, 0.5, 5 and 10 μM), SAHA (10 μM), and vehicle (DMSO) for 24 h. Cells were lysed and the levels of the acetyl-alpha-tubulin was monitored by immunoblotting with the specific antibody. c: control; V: SAHA/vorinostat.

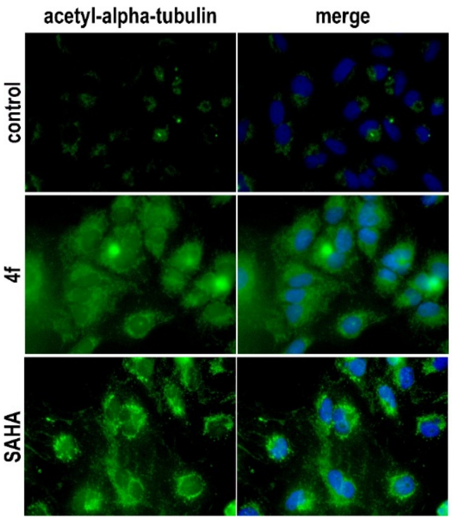

Figure S4. Increasingly acetylated microtubules (acetyl-alpha-tubulin; green) in 518A2 melanoma cells after 24 h treatment with vehicle (control; DMSO), 4f (4 μM), and SAHA (10 μM) as visualized by immunofluorescence microscopy (400× magnification). Nuclei were counterstained with DAPI (merge, blue).

Effect on migratory activity.

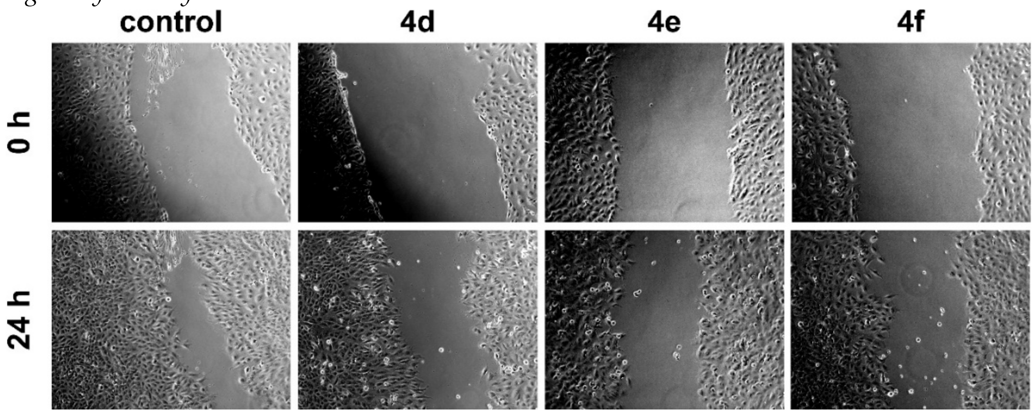

Figure S5. Inhibitory effect of 4d (0.2 µM), 4e (1.2 µM), 4f (3.8 µM) on cell migration of 518A2 melanoma cells. Pictures are representative of at least three independent experiments. Control cells were treated with DMSO only.

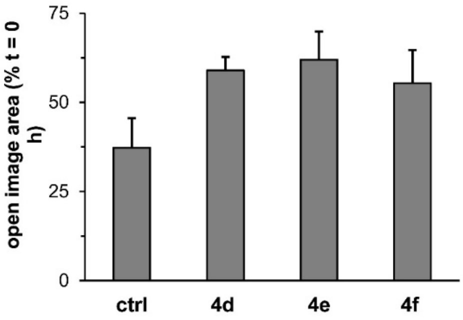

Figure S6. Inhibitory effect of 4d (0.2 µM), 4e (1.2 µM) and 4f (3.8 µM) on the cell migration of 518A2 melanoma cells as determined by wound-healing assays. Values are the means ± SD of at least three independent experiments. Control cells were treated with DMSO only.

Effect on mouse weight.

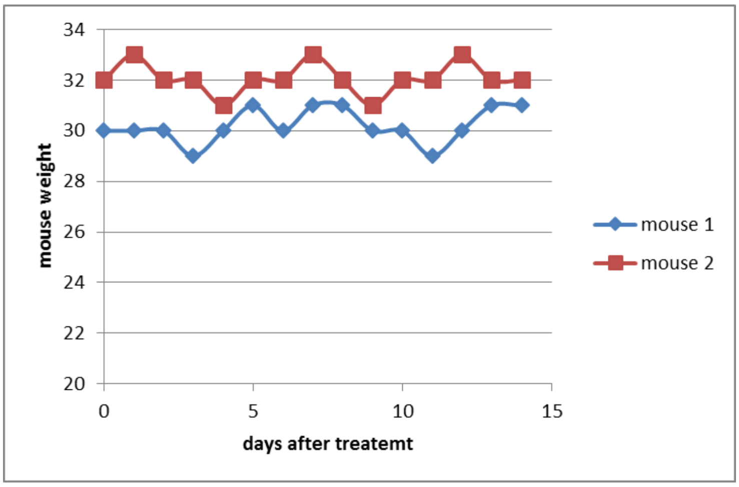

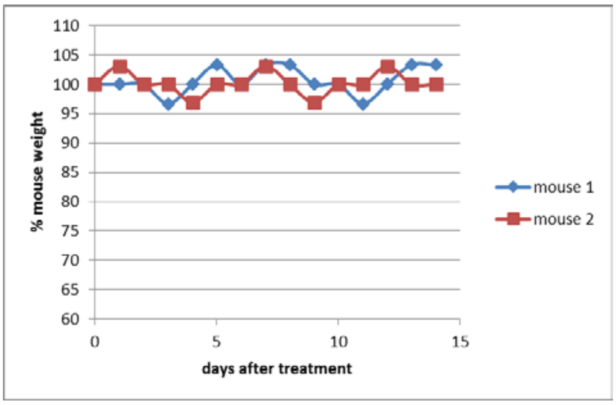

Figure S7. Effect of 4d on mouse weight. One mouse (1) was treated with 1 x 100 mg/kg body weight (i.p.), another mouse (2) was treated with 200 mg/kg (orally).

Graphs of MTT assays.

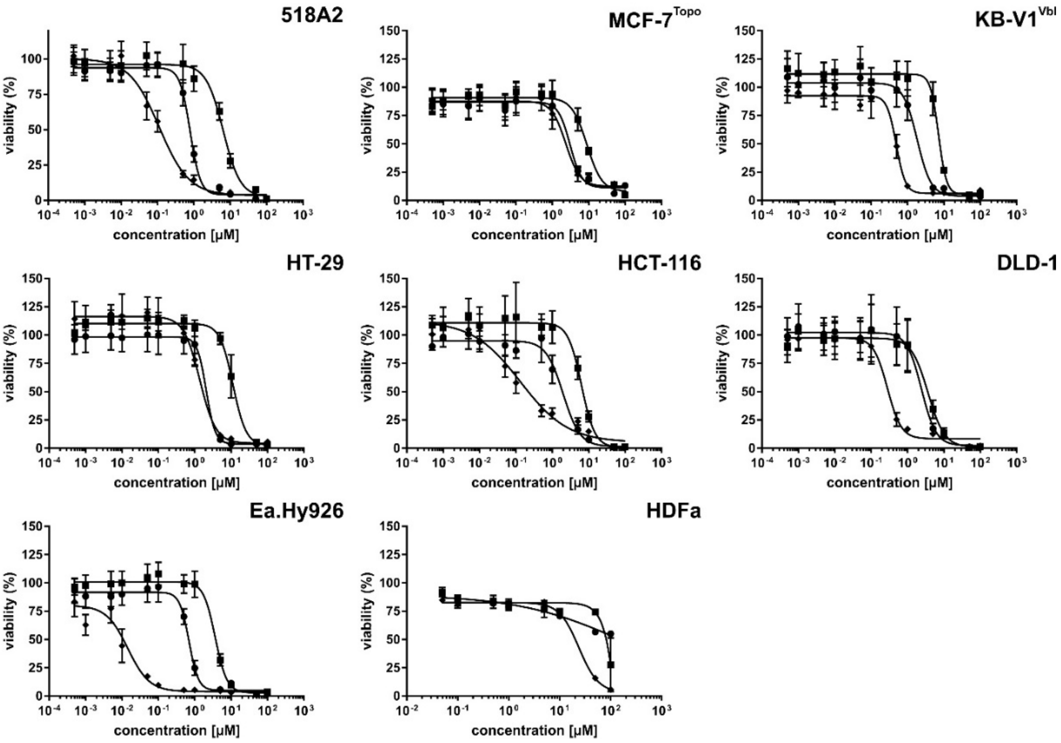

Figure S8. Graphs of compounds 4d-f from MTT assays.
